# Spatial Metabolomics and Lipidomics Reveal the Mechanisms of the Enhanced Growth of Breast Cancer Cell Spheroids Exposed to Triclosan

**DOI:** 10.1021/acs.est.3c01746

**Published:** 2023-07-11

**Authors:** Jing Chen, Peisi Xie, Pengfei Wu, Zian Lin, Yu He, Zongwei Cai

**Affiliations:** † Ministry of Education Key Laboratory of Analytical Science for Food Safety and Biology, Fujian Provincial Key Laboratory of Analysis and Detection Technology for Food Safety, College of Chemistry, Fuzhou University, Fuzhou, Fujian 350116, China; ‡ State Key Laboratory of Environmental and Biological Analysis, Department of Chemistry, Hong Kong Baptist University, Kowloon 999077, Hong Kong SAR, China; § College of Forestry, Nanjing Forestry University, Nanjing, Jiangsu 210018, China

**Keywords:** triclosan, breast cancer cell spheroids, enhanced growth, metabolites and lipids, mass spectrometry imaging

## Abstract

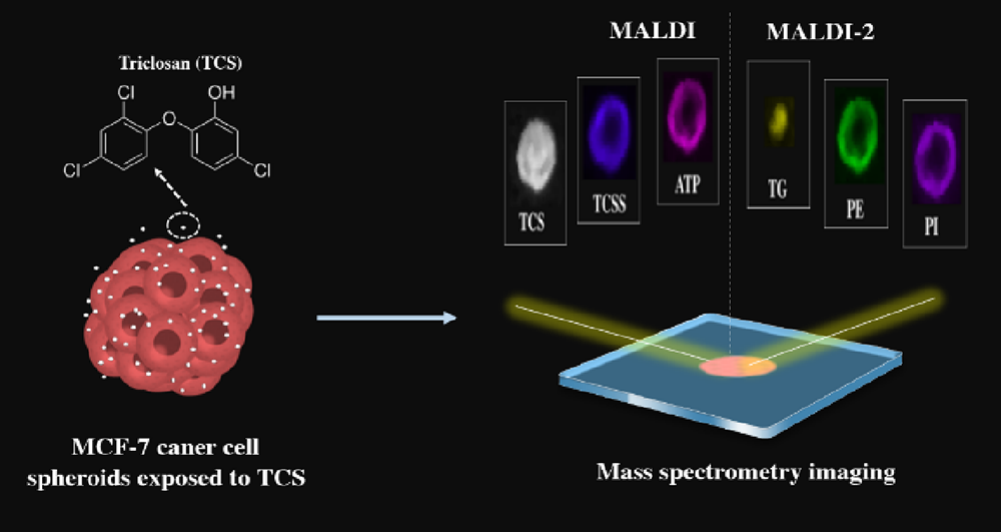

Triclosan (TCS), an antimicrobial compound, is known to have potential endocrine-disruptive properties, but the underlying toxic mechanisms at the metabolic level are not well understood. Here, we applied metabolomics and lipidomics combined with mass spectrometry imaging (MSI) to unveil the mechanisms of the enhanced growth of MCF-7 breast cancer cell spheroids (CCS) exposed to TCS. To obtain a wide coverage of metabolites and lipids by using MSI, we used techniques of matrix-assisted laser desorption/ionization (MALDI) and MALDI coupled with laser-postionization. The results showed that TCS and TCS sulfate penetrated into the entire area at 0–3 h and both localized in the inner area at 6 h. After 24 h, a portion of two compounds was released from CCS. Omic data indicated that TCS exposure induced alterations via several pathways, including energy metabolism and biosynthesis of glycerophospholipids and glycerolipids. Further MSI data revealed that the enhancement of energy supply in the peripheral area and the increase of energy storage in the inner area might contribute to the enhanced growth of MCF-7 breast CCS exposed to TCS. This study highlights the importance of integrating metabolite distributions and metabolic profiles to reveal the novel mechanisms of TCS-triggered endocrine disrupting effects.

## Abstract 2

Minimal research exists on exploring the mechanisms of triclosan-induced endocrine disrupting effects at metabolic levels. This study fills this gap by using a triclosan-exposed breast cancer cell spheroid model.

## Introduction

Triclosan (TCS) is an antimicrobial additive used in over 2000 industrial and consumer products, resulting in being frequently detected in various human samples, including blood, urine, and breast milk.^1^ One previous work suggested that the daily triclosan intake by humans from using products containing triclosan was estimated to be 0.047–0.073 mg/kg/day.^2^ Other studies indicated that the plasma concentration of TCS was 89.7–1021.2 nM following the regular use of consumer products containing TCS, such as toothpaste.^3,4^ TCS can be accumulated in human body. According to previous studies,^5,6^ in human blood, it takes about 12 h for half of the TCS in the blood to be eliminated; in human urine, TCS can be detected up to 8 days after exposure, with peak levels usually occurring within the first 24–48 h; and in human breast milk, TCS can even persist for several weeks. After entering the human body, TCS can undergo two-phase metabolism reactions to combine with sulfate or glucuronic acid, forming TCS-sulfate or TCS-glucuronide.^7^ It can also undergo phase I metabolism reactions to add a hydroxyl group to the phenyl ring, forming OH-TCS.^7^ Although TCS is banned from use in soap products in 2016 by the USA Food and Drug Administration, it still can be used in many other products such as cosmetics, toys, mouthwash, and toothpaste.^8^

Because of its ubiquitous characteristics, there is a growing concern over its impact on human health. One of the important concerns is its endocrine-disrupting effects through interfering with the production, metabolism, and transportation of estrogen in the human body by binding to human estrogen receptors.^9^ Estrogen receptors are proteins that bind to estrogen hormones and are found in many human cells (e.g., breast cells).^10^ The binding of estrogen to these receptors can promote cell growth and division, increasing the risk of cancer (e.g., breast cancers).^10^ Epidemiological studies indicated that estrogen receptor status is a significant risk factor for breast cancer and should be considered in the development of risk prediction models.^11^ One recent cohort study suggested that TCS in human urine has a significant association with breast cancer incidence, indicating that exposure to TCS may elevate the risk of developing breast cancer.^12^*In vitro* studies suggested that TCS exposure could promote the growth, invasion, and migration of MCF-7 breast cancer cells through multiple pathways, including inhibiting the production of reactive oxidative stress (ROS) and endoplasmic reticulum stress, and promotion of the epithelial-to-mesenchymal transition in cells.^13,14^*In vivo* studies showed that TCS exposure could promote the progression of MCF-7 breast tumors by the estrogen receptor-mediated signaling pathway in the xenograft mouse model.^14,15^ Yoon and Kwack performed the gene-expression profiling of the uterus in a TCS-exposed rat model.^16^ They found that TCS exposure induced the upregulation of pathways related to sterol/steroid and hexose metabolic processes and the downregulation of pathways associated with oxidation reduction and extracellular matrix organization. However, little is known about the underlying mechanisms of endocrine-disrupting effects of TCS at the metabolic level.

Metabolomic analysis based on mass spectrometry (MS) is an effective strategy for the assessment of toxic risks by monitoring endogenous metabolites in biological systems that respond to external stresses.^17^ As a metabolomic branch, lipidomic analysis is also an important method for investigating the biological response based upon the analysis of lipid profiles in biological specimens.^18^ Until now, the responses of endogenous metabolites in breast cancer cells exposed to TCS have not yet been well characterized. The combination of lipidomics and metabolomics should be able to provide a comprehensive and detailed understanding of metabolic mechanisms underlying TCS-induced endocrine disrupting effects.

In environmental toxicological studies, two-dimensional (2D) cancer cell models have been widely utilized due to their advantages of low cost and easy operation.^19^ However, 2D cancer cells cannot mimic the three-dimensional (3D) internal structure observed in human solid tumors, which may lead to an inaccurate evaluation of the effects of environmental contaminants on tumor progression. Cancer cell spheroids (CCS) are 3D *in vitro* cell models that are able to more closely mimic many characteristics of solid tumors, such as growth kinetics, gene expression levels, spatial structures, and metabolic characteristics.^20^ With the increasing of CCS diameters (>500 μm), three areas (the inner necrotic area, peripheral proliferative area, and middle quiescent area) would form within CCS due to the decreased permeability of oxygen and nutrients.^20^ Consistent with this, solid tumors also have similar interior structures that include the necrotic layer comprising dead cells and the parenchyma layer comprising quiescent and proliferative cells.^20^ Besides, previous studies demonstrated that, in a hypoxic environment of the inner area of CCS, tumor cells could convert pyruvate to lactate.^20,21^ The accumulation of lactate is a key factor in the acidification of the inner area of CCS, which is also observed in solid tumors.^20,21^ Our recent study also showed that, compared to 2D HepG2 liver cancer cells, 3D liver CCS had more shared lipid species and more similar lipid distribution with solid tumors.^22^ Hence, CCS have gained increasing interests as reliable 3D cell models to better evaluate effects of environmental pollutants on cancer progression.^23,24^ However, no studies have applied 3D breast cell models to evaluate the endocrine-disrupting effects of TCS.

Matrix-assisted laser desorption/ionization mass spectrometry imaging (MALDI-MSI) is a powerful label-free technique that can simultaneously acquire the information of spatial distribution and abundance for different compounds (e.g., metabolites, lipids, and environmental pollutants) in different biological samples.^25,26^ However, MALDI suffers from poor ionization efficiencies for many different analysts. Its estimated ionization efficiencies (the ratio of the number of generated ions to the number of desorbed neutrals) are 10^–4^ and lower.^27,28^ Several approaches have been developed to improve the ionization efficiency of MALDI.^29,30^ The most common approach is to introduce specific functional groups to target analytes by chemical derivatizations. For instance, Girard’s reagents that are quaternary ammonium salts with hydrazine groups could react with a carbonyl group to form hydrazones. By using this strategy, Zecchi et al. successfully developed the method of on-tissue derivatization to detect the spatial distribution of corticosteroids in the animal lung lobes.^31^ However, chemical derivatization has its drawbacks such as a long reaction time and possible analyte delocalization in biological samples.^32^ An alternative method for increasing the ionization efficiencies of MALDI is the application of laser-based postionization strategies, called MALDI-2. These strategies use an additional ionization event, which is temporally and spatially separated from the initial laser-desorption event, to raise the total amount of ions produced.^33^ Unlike chemical derivatization, MALDI-2 does not need additional sampling steps, and it can be carried out directly on the sample used for the traditional MALDI analysis. MALDI-2 was reported to be able to drastically enhance the intensity for multiple compounds (e.g., many lipids, steroids, and drugs).^30,34^ However, this novel method has not been applied into environmental toxicology studies.

Therefore, the goal of this work was to use spatial metabolomics and lipidomics that are composed of techniques of MALDI-MSI and MS-based omics to explore the related mechanisms of TCS-triggered endocrine disrupting effects. Breast CCS produced by MCF-7 human breast tumor cells were chosen as the 3D cell model. This cell line has been widely used in breast cancer research due to its estrogen and progesterone receptor-positive phenotype, which makes it a good model for studying the endocrine-disruptive effects of different environmental contaminants.^13−15^ For the investigation of the distribution and metabolism of TCS in breast CCS, imaging and quantitative analyses of TCS and its metabolites in CCS at various time points by applying MALDI-MSI and liquid chromatography–tandem mass spectrometry (LC–MS/MS) were performed. The metabolic responses of CCS exposed to TCS were evaluated by using MS-based metabolomics and lipidomics. MALDI and MALDI-2 MSI were performed to obtain a wide coverage of spatial distributions of metabolites and lipids in breast CCS. For proof that the accumulation of triglycerides (TG) in the necrotic region of CCS was not associated with cell deaths but related to CCS growth, the expression analysis of genes associated with ROS and inflammation and the treatment of orlistat (an inhibitor of fatty acid synthase) were carried out. Our work may provide a new insight into mechanisms of TCS-induced endocrine disrupting effects.

## Experimental Section

### Chemical and Reagents

Chloroform, acetonitrile (ACN), methanol (MeOH), ammonium acetate, ethanol (EtOH), dichloromethane (DCM), formic acid, and isopropanol (IPA) were from Merck (Darmstadt, Germany). Trypsin (0.25%), Dulbecco’s modified eagle medium (DMEM), dimethyl sulfoxide (DMSO), 4-chloro-phenylalanine (4-Cl-Phe), fetal bovine serum (FBS), collagen I, and penicillin–streptomycin (100 U/mL) were from Thermo Fisher (Cambridge, MA, U.S.A.). *trans*-2-[3-(4-*tert*-Butylphenyl)-2-methyl-2-propenylidene]malononitrile (DCTB), 2,5-dihydroxybenzoic acid (DHB), orlistat, and 9-acridinamine (9AA) were from Sigma-Aldrich (St. Louis, MO, U.S.A.). Phosphatidylcholine(19:0/19:0) (PC(19:0/19:0)), triglyceride(15:0/15:0/15:0) (TG(15:0/15:0/15:0)), and sphingomyelin(d18:1/12:0) (SM(d18:1/12:0)) were obtained from Avanti Polar Lipids (Alabaster, AL, U.S.A.). TCS glucuronide (TCSG, 95%) and TCS sulfate (TCSS, 95%) were purchased from Santa Cruz Biotechnology (Dallas, TX, U.S.A). TCS (99%) was brought from Alfa Aesar (Haverhill, MA, U.S.A). ^13^C_12_-TCS was purchased from Cambridge Isotope Laboratories (Andover, MA, U.S.A).

### Preparation of Breast CCS and TCS Exposure

The MCF-7 breast cancer cells (ATCC, Manassas, U.S.A.) were grown in a DMEM complete medium (without phenol red) containing 89% DMEM, 10% FBS, and 1% penicillin–streptomycin. Breast CCS were cultured in ultralow attachment 96-well plates (Corning Inc., ME, U.S.A.). On day 0, the complete medium (100 μL) containing 3000 cells and collagen I (6 μg/mL) were added into each well of the plates. Plates were centrifuged at 1000 rpm for 3 min and put into an incubator at 37 °C with 5% CO_2_. On day 1, additional 100 μL of complete medium was added into each well and centrifuged at 1000 rpm for 3 min. The plates were put back into the incubator with the same culture condition.

For the examination of the effect of TCS exposure on MCF-7 CCS, on day 5, the medium was completely changed by a fresh medium (200 μL) containing various concentrations of TCS. The final concentrations of TCS in nine groups were 0, 0.05, 0.1, 0.2, 0.5, 1, 2, 5, and 10 μM. The highest exposure level of TCS (10 μM) is lower than the concentration (13 μM) found in human urine.^35^ Besides, most of the exposure levels are also comparable to that (1.02 μM) found in human plasma.^3^ For the investigation of the effect of the accumulation of TG on CCS, MCF-7 CCS were treated with 3 μM of orlistat or a combination of 3 μM of orlistat and 2 μM of TCS on day 5. Each group contained eight replicates. The final concentration of DMSO in each group was 0.1%. Every 72 h, the medium was replaced by 100 μL of fresh medium with various concentrations of TCS. The areas of CCS were measured by an inverted fluorescence microscope at 10× magnification every 72 h. On day 14, each cell spheroid was rinsed with phosphate-buffered saline (PBS) for three times and digested with 0.25% trypsin (100 μL). An automated cell counter was used to measure the cell number for each cell spheroid. Eight replicates were performed for each group.

### Extraction of Metabolites and Lipids

Breast CCS were grown in a DMEM complete medium containing 0.1% DMSO or TCS (2 μM) from day 5 to day 14 in culture. On day 14, breast CCS in control and exposure groups were collected in 1.5 mL tubes and washed with PBS three times. Nine sample replicates were included in each group. Each sample replicate contained 20–30 CCS. The cooled solution (375 μL of 80% MeOH) was added into each sample. CCS were crushed by using a cell sonicator and underwent three freeze–thaw cycles by using liquid nitrogen. A total of 225 μL of chloroform was added followed by shaking and adding 75 μL of deionized water. The tubes were vortexed (1 min) and left (5 min) at room temperature and centrifuged (12,000 rpm, 15 min) at 4 °C. Three layers including the upper layer containing metabolites, the middle layer containing protein, and the bottom layer containing lipids were formed and collected into different tubes. The liquids of metabolites and lipids were dried at 4 °C by using a freezer drier. The assay of bicinchoninic acid was performed to measure the protein content of each sample. The metabolite and lipid residues were dissolved in 50% MeOH (100 μL) containing 1 μg/mL of 4-Cl-Phe and 100 μL of IPA/ACN/water (30:65:5, v/v/v) containing 1 μg/mL of SM(d18:1/12:0), PC(19:0/19:0) and TG(15:0/15:0/15:0), respectively. The solution was sonicated (2 min), vortexed (1 min), and centrifuged (12,000 rpm, 10 min) at 4 °C. Supernatants (80 μL) were collected for further metabolite and lipid analyses.

### LC–MS/MS Instrumental and Data Analyses

A UPLC system coupled with an Orbitrap Fusion Tribrid Mass Spectrometer (Thermo Fisher Scientific, U.S.A.) was used for the analyses of metabolites and lipids in breast CCS. The detailed analytical protocols were described in our previous published works.^25,26^ Briefly, an amide and C18 column were used to separate metabolites and lipids, respectively. The sample temperature was set at 4 °C. The column temperatures were 40 and 50 °C for the separation of metabolites and lipids, respectively. The injection volume was 10 μL. Other conditions including the mobile phase, LC gradient, and MS parameters are listed in Tables S1 and S2. A total of six blank samples and two quality control (QC) samples were arranged into the onset of the running sequence. Besides, one QC sample was included within every six samples.

Softwares of R (version 4.1.1) and Lipidsearch (version 5.0) were utilized to extract ion peaks and align the profiling data of metabolites and lipids, respectively. 4-Cl-Phe was used as the internal standard for analyzing metabolites. Three internal standards ((SM(d18:1/12:0), TG(15:0/15:0/15:0), and PC(19:0/19:0)) belonging to three lipid categories ((sphingolipids (SPs), glycerolipids (GLs), and glycerophospholipids (GPs)) were used as internal standards for analyzing different lipid categories. All the extracted peak areas of metabolites and lipids were normalized by the peak areas of internal standards and protein contents. All significantly changed compounds were selected based on the threshold of *p* value (*p* < 0.05), the score of the variable importance in projection (>1), and the fold change (FC, FC > 1.2 or < 0.8). This chosen threshold is biologically meaningful and relevant according to prior studies.^7,36^ These compounds were identified and manually checked by matching MS and MS/MS information of raw data with three online databases (HMDB and METLIN for metabolites and Lipidsearch for lipids). The tolerance values for product and precursor ions were all 5.0 ppm. The enrichment and partial least-squares discriminant analyses (PLS-DA) were performed by using MetaboAnalyst 5.0. All the final data were presented as mean ± standard error mean (SEM).

### Quantitative Analysis of TCSS, TCSG, and TCSS in the Culture Medium and CCS

On day 11 in culture, cell spheroids were exposed to 2 μM of TCS in 100 μL of culture medium at various time points (0, 1, 3, 6, 12, 24, 48, and 72 h). Each time point contained six replicates. Each replicate included 10–15 CCS or 1–1.5 mL of culture medium. The method to extract TCS, TCSG, and TCSS in CCS was the same as that of endogenous metabolites in CCS without adding chloroform. For the extraction of TCS, TCSG, and TCSS in the cell culture medium, samples in 2.0 mL tubes were centrifuged (5 min, 12,000*g*) at 4 °C. The supernatant was harvested and freeze-dried in a vacuum freeze dryer. A total of 250 μL of MeOH containing 100 ppb of ^13^C_12_-TCS was used to dissolve the residue. The solution was centrifuged (5 min, 12,000*g*) at 4 °C, and the supernatants (100 μL) were used for further quantitative analysis by using a UPLC system coupled to a TSQ Quantiva Triple Quadrupole Mass Spectrometer. The detailed instrumental protocols are listed in Table S3.

### Sample Preparation for MSI Analysis

For the examination of time-dependent penetrations of TCSS and TCS in breast CCS, CCS on day 11 was grown in the complete DMEM medium containing 2 μM of TCS for 0, 1, 3, 6, 12, 24, 48, and 72 h. Each time points contained three replicates. For the investigation of the differences in spatial distributions of metabolites and lipids in CCS between two groups, breast CCS were cultured in the complete DMEM medium with or without TCS (2 μM) from day 5 to day 14 in culture. These CCS were collected and used for further MSI analysis. The detailed protocol of sample preparation was described in our previous work.^15^ Briefly, CCS were washed with normal saline, embedded into gelatin solution (175 mg/mL in deionized water), and preserved at −20 °C for 2 h. The frozen sections (10-μm thickness) of CCS were made by a freezing microtome, thaw-mounted on indium tin oxide (ITO) slides, and stored in a vacuum desiccator. Further MSI studies were performed on CCS sections in the central part of breast CCS.

A DCTB matrix (5 mg/mL in 70% MeOH and 30% DCM) was used to detect TCS and TCSS in breast CCS in negative ionization mode according to the previous work.^25^ DHB (15 mg/mL in 70% MeOH) and 9AA (5 mg/mL in 70% MeOH) matrixes were used to detect endogenous metabolites and lipids in CCS in positive and negative ionization modes, respectively. These matrixes were sprayed on CCS sections by using a commercial instrument, HTX H5 sprayer (HTX Technologies, U.S.A.). The following instrumental parameters were used: flow rate (0.03 mL/min), velocity (2000 mm/min), tracking spacing (2 mm), pressure (10 psi), drying time (20 s for 9AA and DHB, 10 s for DCTB), temperature of spray head (66 °C for 9AA and DHB, 50 °C for DCTB), and the number of spray cycle (16 cycles).

### Acquisition and Analysis of MSI Data

The instrument, timsTOF flex MALDI-2 (Bruker Daltonics, Germany) was used to carry out MSI experiments and was calibrated by using the agilent tuning mixes. The data were acquired at a maximum rate (10,000 Hz for MALDI and 1000 Hz for MALDI-2) within the detection range from *m*/*z* 100 to 1050 in both negative and positive ionization modes. The size of laser spot was set to 20 μm in the single mode. Main-optimized instrumental parameters including funnel 1 RF (350 Vpp), funnel 2 RF (500 Vpp), multipole RF (1200 Vpp), collision RF (1000 Vpp), transfer time (70 μs), prepulse storage (11 μs), laser power (95%), and laser shots (350 shots for MALDI and 50 shots for MALDI-2) were used in this study.

SCiLs Lab 2022a was used to analyze the MSI raw data. The method of total ion count (TIC) and weak denosing were used to normalize spectra and process all ion images, respectively. Segmentation analysis was performed to distinguish different areas in sections of CCS between the blank and exposure groups. The probabilistic latent semantic analysis (pLSA) was implemented to differentiate metabolic differences between two groups across the full area, necrotic area, and proliferative area of CCS. The paired *t* test was carried out to do the statistical comparison of the average intensities of different metabolites and lipids in nine sections from three breast CCS in each group. Metabolites and lipids were assigned at a mass accuracy of less than 5 ppm. Significantly changed compounds were selected based on the *p* value (*p* < 0.05) and fold change (FC, FC > 1.2 or < 0.8). Most of these compounds were further identified by MALDI-MS/MS.

### Gene Expression Analysis and Determination of TG in MCF-7 CCS

RNA in CCS between control and exposure groups was extracted by using TRIzol Reagent (Invitrogen, USA). Each group contained six replicates. Each replicate included 30 CCS. A S1000TM Thermal Cycler (Thermal Scientific, USA) was utilized to convert RNA (450 ng) into cDNA by using the PrimeScriptRT reagent kit (TaKaRa, Japan). A QIAquant 384 5plex system (QIAGEN, Germany) was utilized to perform the quantitative real-time polymerase chain reaction (RT-PCR) using the SYBR Premix Ex Taq kit (Takara, Japan). A total of seven genes including glutathione reductase (GR), nuclear factor-E2-related factor2 (Nrf2), glutathione peroxidase 1 (GPX1), interleukin-6 (IL-6), interleukin-1β (IL-1β), tumor necrosis factor-α (TNF-α), and glyceraldehyde-3-phosphate dehydrogenase (GAPDH) were analyzed. ΔCt was calculated by the difference between the target gene and GAPDH. 2^ΔCt^ was used to represent the level of gene expression.

Levels of TG in MCF-7 CCS were measured among control, orlistat-treated, and the combination of TCS- and orlistat-exposed groups using a TG Assay kit (Jiancheng Bioengineering Institute, Nanjing, China) based on the method of glycerol-3-phosphate oxidase peroxidase-aminophenazone. Briefly, CCS were washed with PBS three times and crushed by using a cell sonicator. Levels of TG in the homogenates were determined according to the instructions of the manufacturer. Each group included six replicates. Each replicate contained 30 CCS.

## Results and Discussion

### Breast CCS Growth after TCS Exposure

**Figure 1:**
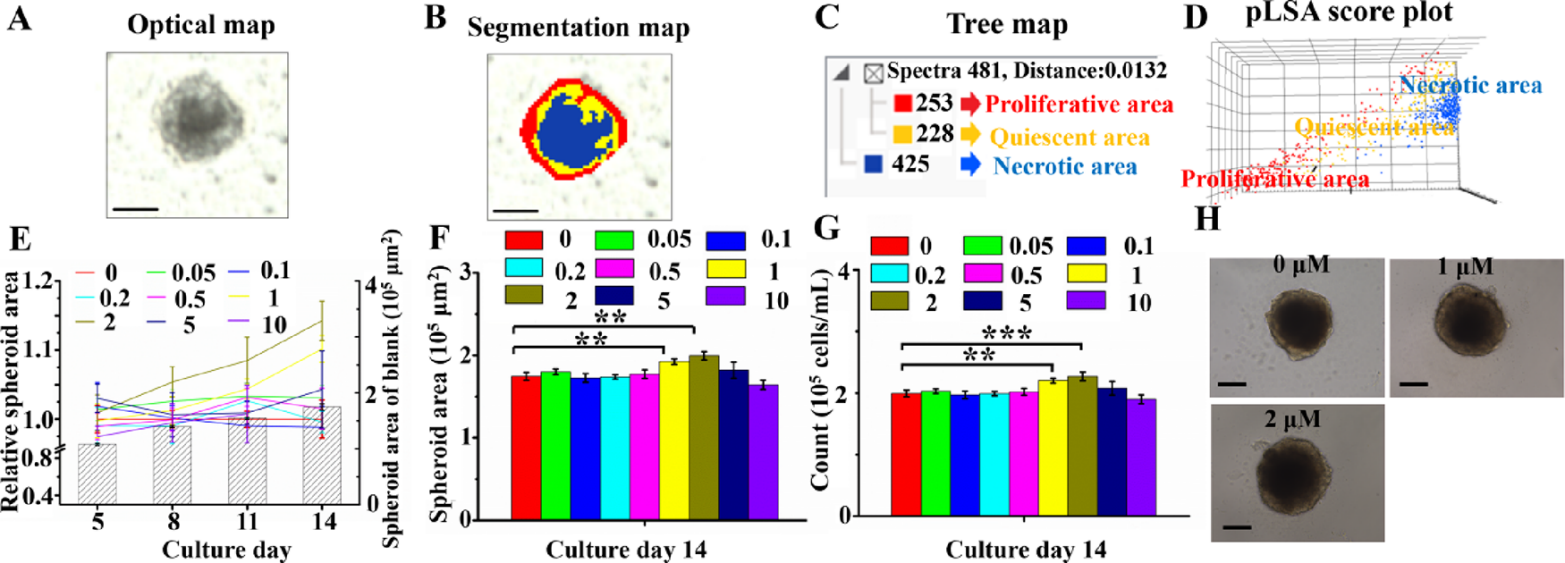
Optical map (A), segmentation map (B), tree map (C), and pLSA score plot (D) of one middle frozen section of one MCF-7 breast cell spheroid grown on day 14 in culture. (E) Growth curves of MCF-7 CCS exposed to TCS at different concentrations (*n* = 8). The areas (F) and the cell number (G) of breast CCS exposed to TCS with different concentrations on day 14 in culture (*n* = 8). (H) Representative images of breast CCS exposed to TCS (0, 1, and 2 μM). All scale bars were 200 μm.

In this work, the estrogen-responsive (ER) positive cell (MCF-7 breast cancer cell) was chosen to establish the 3D cell model to test the endocrine-disrupting effect of TCS. Previous studies reported that there are three areas in CCS due to the limited permeability of nutrients and oxygen.^20^ To prove the successful establishment of MCF-7 breast CCS, we did the segmentation analysis for sections of cell spheroids grown on day 14 in culture. The principle of segmentation analysis is to divide image data into distinct regions or segments based on the chemical composition of the sample. The goal of segmentation analysis is to simplify the MSI data and identify meaningful patterns or trends in the biological sample. The results (Figure 1A,B) showed that the inner necrotic area (blue), the middle quiescent area (yellow), and the peripheral proliferative area (red) were found in breast CCS. The proliferative, quiescent, and inner areas were made up of 253, 228, and 425 spectra, respectively (Figure 1C). The results of pLSA at 95% confidence intervals revealed a clear separation among these three areas (Figure 1D), suggesting that the metabolic characteristics of breast tumor cells in three areas had obvious differences.

We then assessed the effects of TCS on the growth of MCF-7 breast CCS. As illustrated in Figure 1E, with the increasing culture day, CCS areas in the blank group increased steadily. CCS areas in TCS (1 and 2 μM)-exposed groups relative to those in the control group increased gradually. Figure 1F,H shows that the spheroid areas in the 1 and 2 μM TCS-exposed groups were significantly larger than those in the control group. Further analysis of the cell counting (Figure 1G) also demonstrated that cell numbers of TCS (1 and 2 μM)-exposed CCS were more than those of CCS in the control group. Taken together, our results indicated that exposure to TCS (1 and 2 μM) significantly promoted the growth of MCF-7 breast CCS. These results were quite similar to those reported in previous studies.^14,37^ For instance, exposure to 1 μM of TCS induced significant cell proliferations of two ER-positive cells (MCF-7 and VM7Luc4E2 breast cancer cells).^14,37^ The observed effects of 1 μM TCS on the proliferation of both 2D and 3D MCF-7 cancer models could be due to the fact that this concentration is within the range of TCS concentrations that have been shown to have estrogenic activity.^38^ TCS has been shown to bind to estrogen receptors and activate downstream signaling pathways, which can promote cell proliferation.^39^ Besides, as shown in Figure 1F,G, the significantly enhanced growth of CCS was only found in 1 and 2 μM of TCS-exposed groups. This nonmonotonic dose–response phenomenon is frequently seen in researches of endocrine-disrupting effects. For example, VM7Luc4E2 breast cancer cells were exposed to 0.1, 1, and 10 μM of TCS.^37^ The most significant effect of cell proliferation was found in 1 μM of the TCS-exposed group. One possible explanation for our results could be that 1 and 2 μM of TCS may have activated certain signaling pathways that promote CCS growth. Another possible explanation could be that 1 and 2 μM of TCS may have created a more favorable microenvironment for CCS growth. Further studies are needed to confirm these hypotheses and provide a more comprehensive understanding of the dose–response relationship of TCS on CCS growth. Because the most significant effect of the enhanced growth of MCF-7 CCS was found in 2 μM of the TCS-exposed group (Figure 1F,G), 2 μM of the TCS was chosen as the exposure concentration for further MSI and LC analyses.

### Time-Dependent Penetrations of TCS and TCSS in Breast CCS

**Figure 2:**
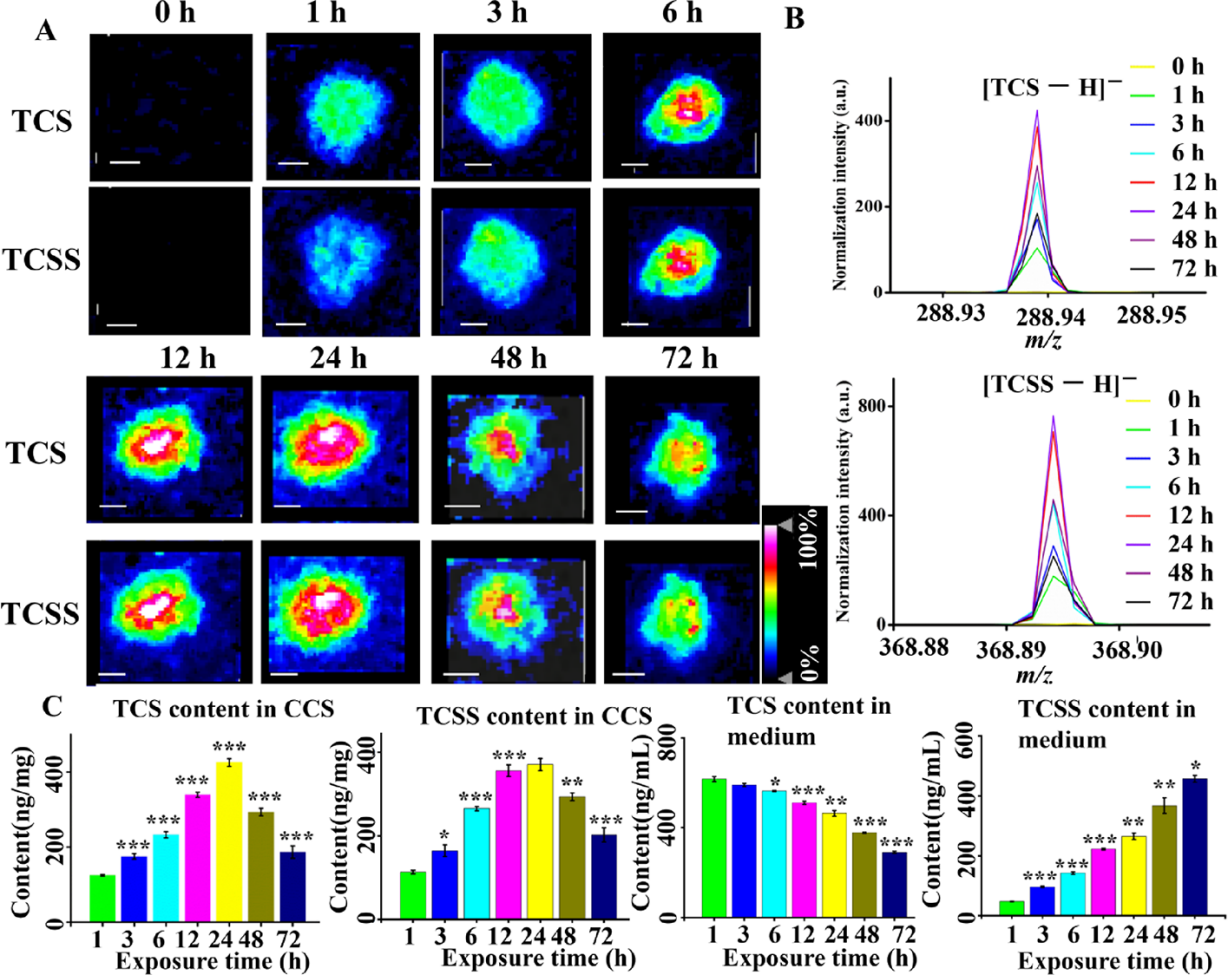
(A) Spatial distributions of TCS (*m*/*z* 288.939) and TCSS (*m*/*z* 368.894) ions in breast CCS exposed to 2 μM of TCS at various time points. The range of intensity values was indicated from 0 to 100%. The color gradient used was a heat map, with white indicating the highest intensity and blue indicating the lowest intensity. The intensity values were normalized to the TIC for each pixel. All scale bars were 200 μm. (B) Representative MALDI-MS spectra of TCS (*m*/*z* 288.939) and TCSS (*m*/*z* 368.894) in CCS exposed to 2 μM of TCS at various time points. (C) TCSS and TCS content in breast CCS and the cell culture medium at various time points. TCS and TCS content in CCS was calibrated by the protein content of CCS. The statistical analysis was carried out between adjacent time points. Data were presented as mean ± SEM. (***p < 0.001, **p < 0.01, *p < 0.05).

Penetrations of environmental pollutants and their metabolites into solid tumors are important for assessing the effects of environmental pollutants on tumor development.^23,40^ Therefore, we applied MALDI-MSI to investigate the distributions of TCS and two phase II metabolites (TCSG and TCSS) in breast CCS exposed to 2 μM of TCS. We chosen 0–72 h as the exposure time range because the culture medium containing various concentrations of TCS was replaced every 72 h. To investigate the time-dependent penetrations of TCS and its metabolites in CCS, the time range was divided into several time points (0, 1, 3, 6, 12, 24, 48, and 72 h). DCTB was used as the MALDI matrix to detect TCS, TCSS, and TCSG in CCS in negative ionization mode according to the previous work.^25^ As shown in Figure 2A and Figure S1A, negligible signals of TCSS and TCS were observed in the unexposed CCS, indicating that no endogenous metabolites were interfered with TCSS and TCS detection. Two major ion peaks (Figure 2B and Figure S1B) were found in both [TCSS – H]^−^ and [TCS – H]^−^ owing to the existence of chlorine-37 and chlorine-35. From 1 to 24 h, TCS penetrated from the full area to the center area of cell spheroids, while TCS distributed from the outer area to the entire area and eventually located in the inner area of cell spheroids. After 24 h, a portion of TCS and TCCS were gradually eliminated from CCS. The representative spectra of TCS and TCSS at different exposure times are presented in Figure 2B and Figure S1B. The results showed that the intensities of TCS and TCSS in CCS gradually increased from 0 to 24 h and decreased after 24 h. In this study, TCSG was not detected in CCS sections at all exposure time points by using MALDI-MSI.

The penetration of TCS in the full area of MCF-7 CCS at 1 h may be because TCS is a hydrophobic compound that can easily enter the cell membrane of spheroids. The fast penetration of TCS may contribute to its combination with estrogen receptors in MCF-7 cells to activate downstream signaling pathways to promote cell proliferation. Once TCS penetrated into breast CCS, a portion of TCS will be metabolized into TCSS by the enzyme (sulfotransferase) in outer proliferative cells. However, TCS in the inner necrotic area that contains dead cells is not able to be metabolized. This may lead to the result that TCS penetrated into the entire area of CCS more quickly than TCSS. Due to the difference in the metabolic rate of cells within two areas (necrotic area < proliferative area) and increasing content of TCS in cell spheroids, TCSS and TCS permeating into the necrotic area may accumulate. Meanwhile, a fraction of TCSS and TCS in the proliferative area may be released into the cell culture medium. This may be contributing to the concentrated distributions of TCS and TCCS in the inner area of CCS after 6 h. The obtained results were consistent with our previous study.^25^ It demonstrated that, when HCT116 colon CCS were exposed to TCS (10 μM), TCSS gradually localized from the periphery area to the full area at 0 to 12 h and accumulated in the inner area after 24 h. With the increasing intensities of TCS and its metabolic wastes (e.g., TCSS) in cell spheroids from 0 to 24 h (Figure 2A,B), the accumulation of TCS and its metabolic wastes may not good for cell spheroids to achieve a fast growth. Hence, the cells in spheroids may tend to eliminate a portion of TCS and metabolic wastes from the spheroids after 24 h.

For a better understanding of TCS metabolism in breast CCS, the content of TCSS, TCSG, and TCS in the culture medium and breast CCS were measured by LC–MS/MS. As depicted in Figure 2C and Figure S2, TCS content in the culture medium gradually decreased, while the gradual increased content of TCSG and TCSS was found in the culture medium. This may be due to the continuous consumption of TCS in CCS and the continuous release of TCSG and TCSS from CCS. The content of TCSG, TCSS, and TCS in breast CCS increased gradually from 1 to 24 h and reduced gradually after 24 h, which was consistent with the MALDI-MSI data. This may be because, before 24 h, the metabolic rate of TCS is lower than its permeating rate, which may result in the elevated TCS content in CCS. However, the metabolic rate of TCS may be higher than its permeating rate after 24 h, resulting in the decreased TCS content in breast CCS. For TCSS, as the TCS content in CCS decreased, the TCSS content released into CSS may be lower than that released into the culture medium, resulting in a decreased TCSS content in CCS.

### Metabolomic and Lipidomic Analyses of Breast CCS Exposed to TCS

**Figure 3:**
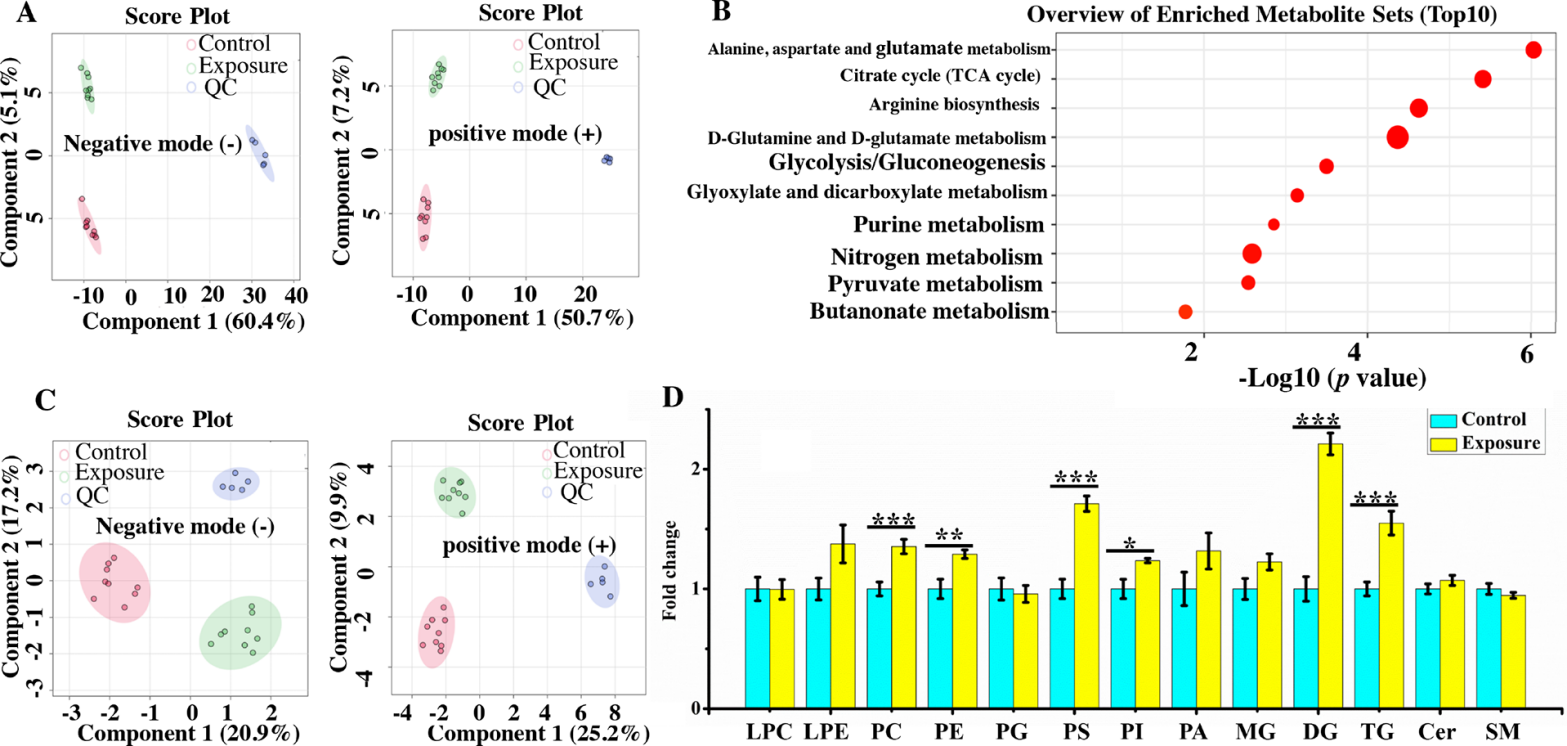
(A) pLSDA analysis of metabolomics in positive and negative ionization modes (*n* = 9). (B) Metabolite set enrichment analysis showing the top 10 altered pathways. (C) The pLSDA analysis of lipidomics in positive and negative ionization modes (*n* = 9). (D) Fold changes of various lipid classes (*n* = 9). Metabolomic and lipidomic analyses were performed in breast CCS between the exposure (2 μM of TCS) and the control groups. Data were presented as mean ± SEM. (***p < 0.001, **p < 0.01, *p < 0.05).

Metabolites such as carbohydrates, amino acids, and lipids act important roles in many biological processes including cell signaling, energy supply, and energy storage.^41^ Prior studies showed that metabolic disorders were closely related to cancer growth and development.^42^ Therefore, to explore the metabolic mechanisms of the enhanced growth of CCS exposed to TCS, we conducted MS-based metabolomic and lipidomic analyses of MCF-7 breast CCS on day 14 in culture between the exposure and control groups. The results of PLS-DA for the metabolomic analysis revealed obvious separations between the exposure and control groups in both negative and positive ionization modes (Figure 3A), indicating that TCS (2 μM) exposure disturbed metabolic levels in breast CCS. The obtained *Q*^2^ values were all above 0.8 in two ionization modes (Figure S3A), indicating a good predictive ability of our metabolomic data. The clustered QC samples suggested a stable instrumental performance (Figure 3A). Levels of 27 metabolites were found to change significantly (Table S4). Among these metabolites, levels of five metabolites including oxaloacetate (FC = 0.38), citrate (FC = 0.55), glyceraldehyde 3-phosphate (FC = 0.66), uridine diphosphate glucuronic acid (FC = 0.69), and carnitine (FC = 0.64) were downregulated, while levels of other 22 metabolites such as ATP (FC = 1.32), ADP (FC = 1.40), fumarate (FC = 1.37), glutamine (FC = 2.25), and glutamate (FC = 1.30) were upregulated. These altered 27 metabolites were further used to perform the metabolite set enrichment analysis. Figure 3B shows the top10 altered pathways, such as alanine, aspartate and glutamate metabolism, tricarboxylic acid (TCA) circle, arginine biosynthesis, d-glutamine and d-glutamate metabolism, and glycolysis. The altered pathways suggested that exposure to TCS may have endocrine-disrupting effects on estrogen signaling pathways in MCF-7 cells. Specifically, the alanine, aspartate, and glutamate metabolism pathway is known to play a role in estrogen receptor signaling and the arginine biosynthesis pathway has been implicated in the regulation of estrogen receptor activity.^43,44^ Additionally, the altered TCA cycle and pyruvate metabolism pathways may affect the availability of energy and substrates for estrogen biosynthesis and signaling. These identified pathways also suggested that TCS exposure may disrupt cellular metabolism and energy production, which can have downstream effects on hormone signaling and other physiological processes.

For the lipidomic analysis of breast CCS, the results of pLSDA (Figure 3C) also showed obvious separations between two groups in both negative and positive ionization modes, indicating the disturbance of lipid metabolism in CCS exposed to 2 μM of TCS. The results of cross validations in two modes (Figure S3B) showed satisfied values of *Q*^2^, suggesting a good predictive ability of the lipidomic data. Levels of 362 lipids were significantly changed. Among these lipid species, 24 lipids belonged to sphingolipids (SPs), including 12 SMs and 12 ceramides (Cers) (Table S5). A total of 162 lipids were glycerolipids (GLs), involving 2 monoglycerides (MGs), 80 diglycerides (DGs), and 80 TGs (Table S5). The rest lipids were glycerophospholipids (GPs), consisting of 1 lysophosphatidylcholine (LPC), 2 lysophosphatidylethanolamines (LPEs), 16 phosphatidylglycerols (PGs), 10 phosphatidylserines (PSs), 12 phosphatidylinositols (PIs), 55 phosphatidylethanolamines (PEs), and 80 PCs (Table S5). Considering that different biological functions are associated with different lipid classes, we investigated the differences in levels of various lipid classes between control and exposure groups. As shown in Figure 3D, levels of PC, PE, PS, PI, TG, and DG were significantly upregulated.

Previous works showed that substantial energy storage and supply are necessary to promote cell growth and proliferation.^45^ Thus, we investigated four main altered pathways (glycolysis, TCA cycle, glutamate metabolism, and biosynthesis of GPs and GLs) associated with energy metabolism and lipid metabolism in breast CCS (Figure S4). Our results demonstrated that TCS exposure led to elevated levels of six metabolites (ATP, ADP, α-ketoglutarate, fumarate, 3-phosphoglycerate, and phosphoenolpyruvate) and reduced levels of three metabolites (glyceraldehyde-3-P, citrate, oxaloacetate) in TCA cycle and glycolysis (Figure S4 and Table S4), suggesting an increasing energy supply in breast CCS after TCS exposure. Another important metabolic characteristic of cancer cells is the enhanced glutaminolysis to produce more energy.^46^ Increased levels of glutamate and glutamine were found in breast CCS exposed to TCS (Figure S4 and Table S4), suggesting the enhanced energy supply from glutamate metabolism. Hence, from the metabolomic data, increased energy supply induced by TCS may contribute to the enhanced growth of breast CCS. In the pathway of biosynthesis of GPs and GLs, PE and PC are main components of mammalian cell membranes. It has been demonstrated that, to obtain rapid cell proliferation, tumor cells have an increased production of PE and PC serving as a building block of the plasma membrane.^47^ PI is required to activate the phosphatidylinositol 3-kinase signaling pathway, which is involved in mediating cell survival and proliferation.^48^ TG and DG are known to act major roles in energy storage. They can be utilized as energy sources under hyperoxic condition by processes of hydrolyzation and β-oxidation.^49^ In this study, elevated levels of DG and TG were observed in TCS-exposed breast CCS, indicating the enhancement of energy storage from lipid metabolism. Taken together, our data suggested that TCS exposure led to the enhanced growth of breast CCS may via increasing energy supply and storage in CCS.

### MALDI and MALDI-2 Broaden the Detection Coverage of Metabolites and Lipids in Breast CCS

**Figure 4:**
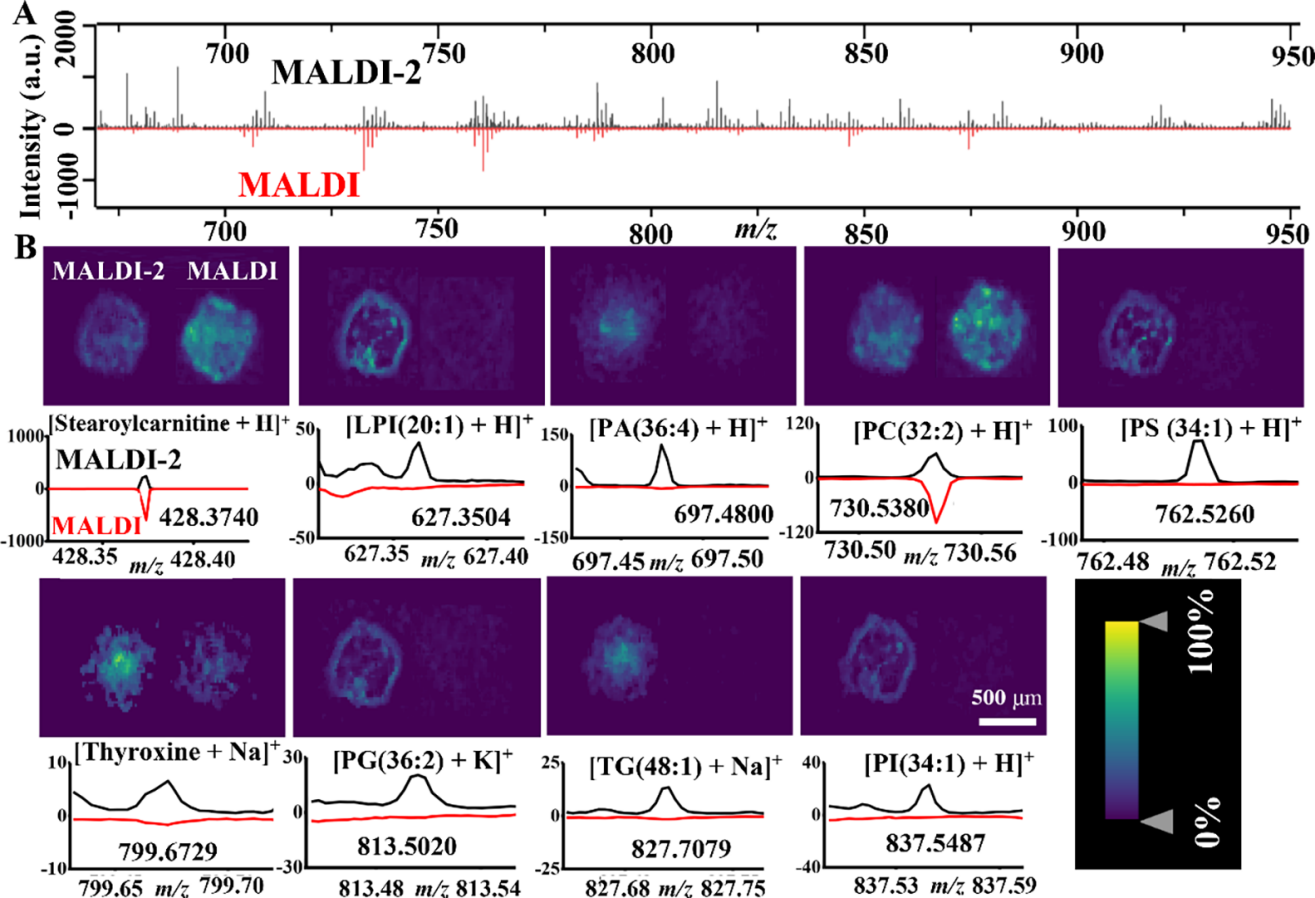
(A) Mass spectra of MALDI-2 (black color) and MALDI (red color) in breast CCS sections in positive ionization mode using the DHB matrix. (B) Representative MALDI-2 images (left) and MALDI images (right) of different ions in breast CCS sections. The corresponding ion spectra of MALDI-2 (upper black color) and MALDI (bottom red color) were listed below the ion images. The range of intensity values was indicated from 0 to 100%. The color gradient used was a heat map, with yellow indicating the highest intensity and blue indicating the lowest intensity. The intensity values were normalized to the TIC for each pixel.

MS-based metabolomics has been frequently used in environmental toxicology, aiming to uncover the underlying metabolic mechanism of action of environmental pollutants.^7^ In this technology, crushing and homogenizing biological samples to extract biomolecules result in the loss of information on molecular spatial distributions. This information might act a major role in clarifying the underlying mechanisms. Therefore, we applied MSI to investigate spatial distributions of endogenous metabolites in CCS. To obtain a wide detection coverage of endogenous biomolecules, we first compared the number and types of detected biomolecules by using MALDI and MALDI-2. DHB and 9AA matrixes were used in positive and negative ionization modes with a detection range from *m*/*z* 100 to 1050. All ion peaks were assigned to endogenous compounds with mass-to-charge ratio less than 5 ppm. As shown in Figure 4A, the spectra of MALDI-2 exhibited more dense peaks than that of traditional MALDI in positive ionization mode. A total of 96 (26 metabolites and 70 lipids) and 64 (32 metabolites and 32 lipids) endogenous molecules were detected by MALDI-2 and MALDI, respectively (Table S6). These metabolites belonged to various types of metabolites, such as 17 fatty acid derivatives (e.g., stearoylcarnitine, cervonyl carnitine), three amino acids (l-carnitine, *N*-a-acetyl-l-arginine, and selenocysteineand), and two coenzymes (coenzyme Q9 and tetrahydrofolic acid) (Table S6). Two coenzymes had higher intensities in MALDI-2 than in MALDI, while 3 amino acids and 15 fatty acid derivatives had lower intensities in MALDI-2 than in MALDI, suggesting that MALDI-2 may showed enhanced ability to detect coenzymes but not amino acids and fatty acid derivatives. A total of 23 metabolites were shared between MALDI and MALDI-2 (Figure S5A). Among these metabolites (Figure 4B and Table S6), intensities of 13 metabolites (e.g., stearoylcarnitine) in MALDI were higher than those in MALDI-2, while 10 metabolites (e.g., thyroxine) had lower intensities in MALDI than in MALDI-2. For lipids, 28 lipids including 2 DGs, 2 LPCs, 5 PEs, 5 SMs, and 14 PCs were shared between MALDI and MALDI-2 (Figure S5B). Compared with MALDI, MALDI-2 was able to detect more lipid classes (Figure 4B and Table S6), such as PI (e.g., PI(34:1)), TG (e.g., TG(48:1)), PG (e.g., PG(36:2)), PS (e.g., PS(34:1)), PA (e.g., PA(36:4)), and LPI (e.g., LPI(20:1)). However, MALDI demonstrated an enhanced ability to detect PC species (e.g., PC (32:2)) compared to MALDI-2 (Figure 4B and Table S6). These results were consistent with several previous reports showing that MALDI-2 was able to increase the intensities for various lipid classes except for PC.^50,51^

In negative ionization mode, intensities of various peaks from *m*/*z* 675 to *m*/*z* 825 in MALDI-2 were higher than those in MALDI, while intensities of peaks over *m*/*z* 825 in MALDI-2 were lower than those in MALDI (Figure S6A). A total of 10 and eight metabolites were detected in MALDI and MALDI-2 (Figure S5C). These metabolites belonged to three types of metabolites including two cyclic nucleotides (inositol cyclic phosphate, ADP-ribose 1′,2′-cyclic phosphate), five fatty acids (e.g., vaccenic acid, stearic acid), and three nucleotides (AMP, ADP, ATP) (Table S7). Among eight shared metabolites between two methods (Figure S5C), six metabolites (e.g., ADP and eicosatrienoic acid) in MALDI had higher intensities than in MALDI-2, while two metabolites (AMP and inositol cyclic phosphate) had lower intensities in MALDI than in MALDI-2 (Figure S6B and Table S7). A total of 40 and 32 lipids were detected in MALDI and MALDI-2, respectively (Figure S5D). Intensities of LPI(18:0), LPA(18:1), and PIs (e.g., PI(38:4)) in MALDI were higher than in MALDI-2, while intensities of PEs (e.g., PE(36:1)) in MALDI were lower than in MALDI-2 (Figure S6B and Table S7). This may be because MALDI-2 involves additional fragmentation of ions during the ionization process. In some cases, the additional fragmentation may result in a loss of sensitivity for certain types of molecules.^52^ Besides, the selection of the matrix may affect the results for detecting compounds by using MALDI-2. For instance, MG(18:1) and CE (18:1) could not be detected in both MALDI-2 and MALDI using the DHB matrix.^51^ However, using the norharmane matrix, these two compounds could be detected in MALDI-2 but not in MALDI.^51^

Taken together, our results showed that, compared with MALDI, MALDI-2 had enhanced or reduced abilities for detecting different metabolites and lipids in different ionization modes. The combination of MALDI and MALDI-2 could broaden the detection coverage of metabolites and lipids in breast CCS. To the best of our knowledge, this is the first work to investigate the spatial distributions of metabolites and lipids in CCS by using MALDI-2 and MALDI MSI.

### MSI Analysis of Breast CCS Exposed to TCS

**Figure 5:**
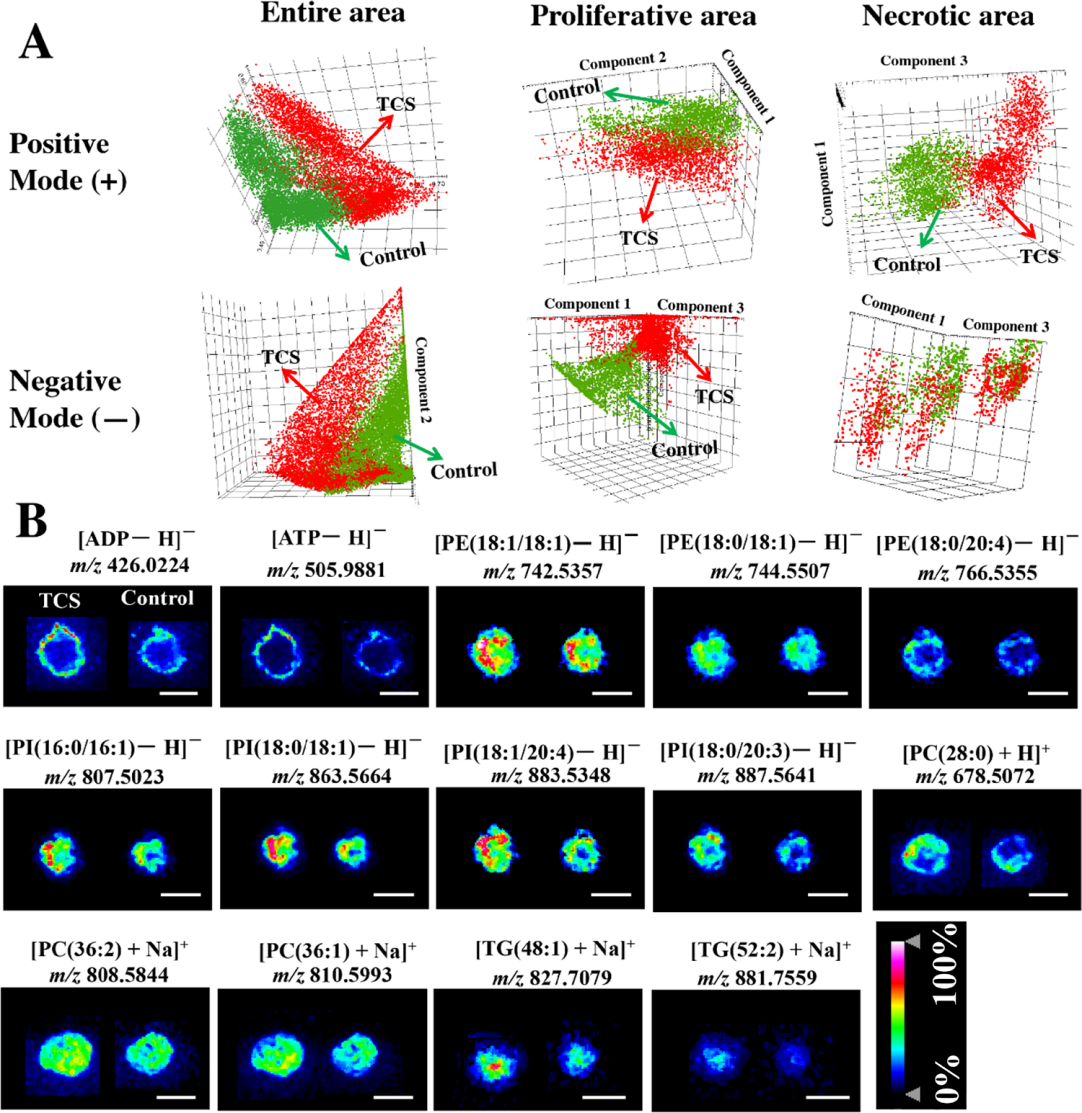
(A) pLSA score plots of MALDI profiles in different CCS areas in positive and negative ionization modes (*n* = 9). (B) Ion images of metabolites and lipids in sections of CCS between TCS-exposed and control groups. The range of intensity values was indicated from 0 to 100%. The color gradient used was a heat map, with white indicating the highest intensity and blue indicating the lowest intensity. The intensity values were normalized to the TIC for each pixel. All scale bars were 500 μm.

The results of mass-based metabolomics and lipidomics (Figure 3) showed the altered levels of several metabolites and six lipid classes (PS, PI, PE, PC, TG, and DG) in breast CCS exposed to TCS (2 μM). To investigate the variations of spatial distributions and abundance of these endogenous compounds in CCS, we performed MSI analysis of CCS sections between exposure and control groups. MALDI-2 and MALDI were used in the positive ionization mode. MALDI was used in the negative ionization mode. The results of pLSA score plots (Figure 5A and Figure S7A) revealed clear separations in three areas (necrotic area, proliferative area, and entire area) of CCS between exposure and control groups in positive ionization modes. However, in negative ionization mode, separations were only found in the proliferative area and entire area, indicating obvious changes in levels of metabolites in these areas of breast CCS after TCS exposure.

The elevated abundance of 33 endogenous molecules including 2 metabolites, 5 PEs, 7 PIs, 13 PCs, and 6 TGs were found in TCS-exposed CCS sections (Table S8). Most of these molecules were identified by using MALDI-MS/MS (Figure S8). Interestingly, two upregulated metabolites, ATP and ADP, were located in the outer proliferative area of CCS, suggesting an increased energy supply in breast CCS to promote cell proliferation after TCS exposure. Four PEs (PE(16:0/20:4), PE(18:1/18:2), PE(18:0/20:4), and PE(18:1/18:1)) mainly located in the periphery area of CCS, while PE(18:0/18:1) located in the entire area of breast CCS (Figure 5B and Figure S7B). All seven upregulated PIs (e.g., PI(16:0/16:1), PI(18:0/18:1), PI(18:1/20:4), and PI(18:0/20:3)) showed high signal intensities in the outer area of breast CCS (Figure 5B and Figure S7B). For PCs, eight lipids (e.g, PC(36:2) and PC(36:1)) mainly distributed in the entire area of CCS, while five lipids (e.g, PC(28:0)) tended to distribute more in the outer region of CCS (Figure 5B and Figure S7B). These results suggested an enhanced biosynthesis of GPs in MCF-7 CCS after TCS exposure, especially in the outer region.

For TGs, interestingly, all detected six lipids (e.g., TG(48:1) and TG(52:2)) using MALDI-2 were predominantly observed in the center area of CCS (Figure 5B and Figure S7B). When energy intake is higher than energy consumption, the extra energy is saved in the form of TGs. They are stored as lipid droplets in cells and are considered to be less toxic.^53^ To prove that the accumulation of TGs was not associated with cell deaths in the necrotic area of CCS, we performed the expression analysis of genes related with inflammation and ROS in CCS between two groups. The results showed that TCS exposure did not alter expression levels of three genes (GR, Nrf2, and GPX1) related to ROS and three genes (IL-6, IL-1β, and TNF-α) associated with inflammation (Figure S9). Moreover, ceramides (Cer) are believed to be lipotoxic and proinflammatory and lead to producing more ROS by triggering mitochondrial oxidative respiration.^53^ In this study, levels of Cer did not show any significant changes between two groups (Figure 3D), suggesting that TCS exposure did not cause the ROS in breast CCS.

Previous studies showed that to achieve a fast growth and proliferation, breast cancer cells require a lot of energy and nutrients.^54^ They can break TG into fatty acids and glycerol to provide ATP and material basis for building cell membranes.^55^ Elevated levels of TGs can increase the availability of lipid substrates and energy to meet the metabolic demands of rapidly dividing cancer cells.^56^ In the tumor microenvironment, TG can serve as an energy reserve for highly aggressive cancers, promoting metastatic growth and progression.^56^ Thus, to prove that the accumulation of TG in CCS may contribute to their growth, we treated MCF-7 CCS with 3 μM of orlistat (one common inhibitor of fatty acid synthase) to reduce the TG levels in CCS. The reason for selecting 3 μM of orlistat as the treatment concentration was that it did not cause cytotoxicity in MCF-7 cancer cells.^57^ The results showed that treatment of orlistat significantly lowered TG levels in CCS (Figure S10D) and inhibit their growth (Figure S10A-S10C). However, when CCS were treated with a combination of 3 μM of orlistat and 2 μM of TCS, we found that TCS alleviated the inhibitory effect of orlistat (Figure S10A-10C) and attenuated its ability to reduce TG levels in MCF-7 CCS (Figure S10D). Taken together, all these results suggested that the enhancement of energy supply in the peripheral area and the increase of energy storage in the inner area might be contributing to the enhanced growth of breast CCS exposed to TCS.

## Supporting Information Available

Time-dependent penetrations of TCSS and TCS in breast CCS; TCSG content in breast CCS and the culture medium at different exposure time; cross validations of pLSDA of metabolomic and lipidomic analyses; metabolic and lipidomic networks; Venn diagrams for metabolites and lipids in positive and negative ionization modes; mass spectra and ion images in MALD-2 and MALDI; pLSA score plots of MALDI-2 profiles and representative ion images; MALDI-MS/MS spectra of endogenous molecules in breast CCS; fold changes of various genes related to reactive oxidative stress and inflammation; the effect of the accumulated triglyceride in MCF-7 breast CCS on their growth; main parameters for the analyses of metabolites, lipids, TCS, TCSS, and TCSG by UPLC-MS/MS; information of significantly changed metabolites and lipids detected by UPLC-MS/MS; information of endogenous metabolites and lipids in positive and negative ionization modes detected by MALDI and MALDI-2 MSI; and information of significantly changed lipids and metabolites identified by MALDI-MSI (PDF)
