# Supplementary material for: Spatial Metabolomics and Lipidomics Reveal the Mechanisms of the Enhanced Growth of Breast Cancer Cell Spheroids Exposed to Triclosan
Source: Environ Sci Technol. 2023 Jul 11;57(29):10542–53. doi: 10.1021/acs.est.3c01746 (PMC10373480; doi:10.1021/acs.est.3c01746)
Supplement: Supplementary file 1 — es3c01746_si_001.pdf [file es3c01746_si_001.pdf]

Spatial metabolomics and lipidomics reveal the mechanisms of enhanced growth of breast cancer cell spheroids exposed to triclosan

Jing Chen[1§], Peisi Xie[1§], Pengfei Wu[2,3], Zian Lin[1], Yu He[1], Zongwei Cai[1,2]*

[1]Ministry of Education Key Laboratory of Analytical Science for Food Safety and Biology, Fujian Provincial Key Laboratory of Analysis and Detection Technology for Food Safety, College of Chemistry, Fuzhou University, Fuzhou, Fujian, 350116, China

[2]State Key Laboratory of Environmental and Biological Analysis, Department of Chemistry, Hong Kong Baptist University, Hong Kong SAR 999077, China

[3] College of Forestry, Nanjing Forestry University, Nanjing, Jiangsu, 210018, China

[§]Both authors contributed equally to this work.

[*]Corresponding author:

Prof. Zongwei Cai, Tel: +852-34117070; Fax: +852-34117348; Email: zwcai@hkbu.edu.hk

This Supporting Information contains 49 pages, 8 tables and 10 figures.

Table of Contents

Figure S1 Time-dependent penetrations of TCSS (*m/z* 366.896) and TCS (*m/z* 286.942) in breast CCS...………..S4

Figure S2 TCSG content in breast CCS and culture medium at different exposure time……………………………………..………………………………………S5

Figure S3 Cross validations of pLSDA of metabolomic and lipidomic analyses…...S6

Figure S4 Metabolic and lipidomic networks...S7

Figure S5 Venn diagrams for metabolites and lipids in positive and negative ionization modes...S8

Figure S6 Mass spectra and ion images in MALD-2 and MALDI............................S9

Figure S7 pLSA score plots of MALDI-2 profiles and representative ion images……………………………………………………………..………. S10

Figure S8 MALDI-MS/MS spectra of endogenous molecules in breast CCS......... S11

Figure S9 Fold changes of various genes related to reactive oxidative stress and inflammation…………………………………………………………….……S16

Figure S10 The effect of the accumulated triglyceride in MCF-7 breast CCS on their growth..……………………………………………………………….……S17

Table S1 Main parameters for the analysis of metabolites by UPLC-MS/MS….....S18

Table S2 Main parameters for the analysis of lipids by UPLC-MS/MS…………...S19

Table S3 Instrumental methods for the quantitative analysis of TCS, TCSS and TCSG by UPLC-MS/MS……………………………………………………..S20

Table S4 Information of significantly changed metabolites detected by UPLC-MS/MS…………………………………………………………………..………S21

Table S5 Information of significantly changed lipids detected by UPLC-MS/MS…………………………………………………………………………..S23

Table S6 Information of endogenous metabolites and lipids in positive ionization mode detected by MALDI and MALDI-2 MSI……………………..…………….……......S39

Table S7 Information of endogenous metabolites and lipids in negative ionization mode detected by MALDI and MALDI-2 MSI…………………...…………….…......S45

Table S8 Information of significantly changed lipids and metabolites identified by MALDI-MSI……………………………………………………………...…..S48

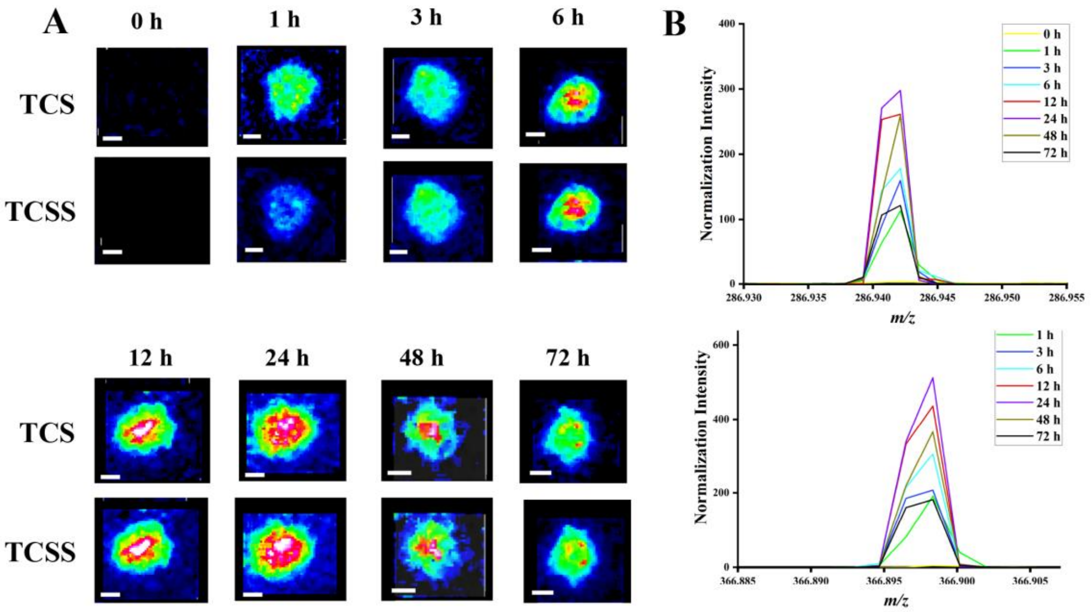

Figure S1. Time-dependent penetrations of TCSS (*m/z* 366.896) and TCS (*m/z* 286.942) in breast CCS. (A) Spatial distributions of TCS (*m/z* 286.942) and TCSS (*m/z* 366.896) ions in breast CCS treated with 2 μM of TCS at various time points. The range of intensity values was indicated from 0 to 100%. The color gradient used was a heat map, with white indicating the highest intensity and blue indicating the lowest intensity. The intensity values were normalized to the total ion count (TIC) for each pixel. All scale bars were 200 μm. (B) Representative MALDI spectra of TCS (*m/z* 286.942) and TCSS (*m/z* 366.896) in CCS exposed to 2 μM of TCS at various time points. Scale bars in all ion images were 200 μm.

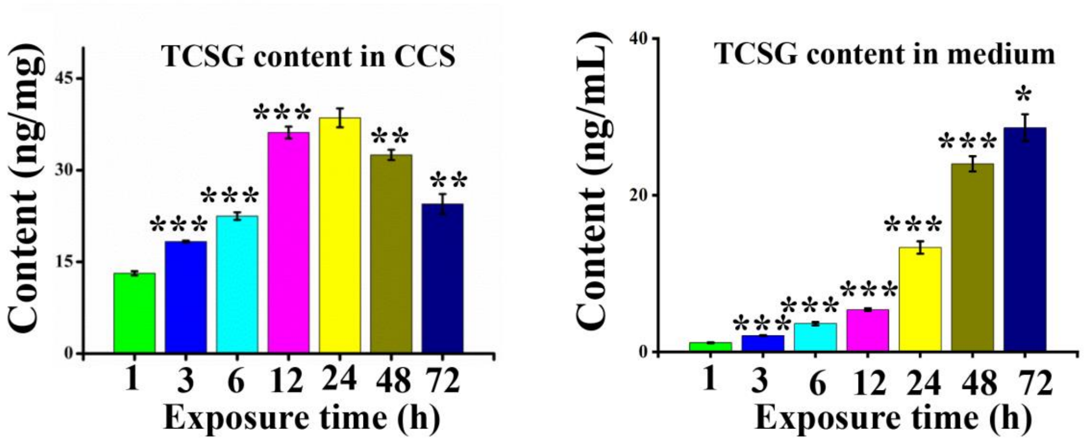

Figure S2. TCSG content in breast CCS and culture medium at different exposure time. CCS were exposed to 2 μM of TCS. TCSG content in CCS was calibrated by the protein content. The statistical analysis between adjacent time points was carried out. The error bars represented SEM. (***p < 0.001, **p < 0.01, *p < 0.05).

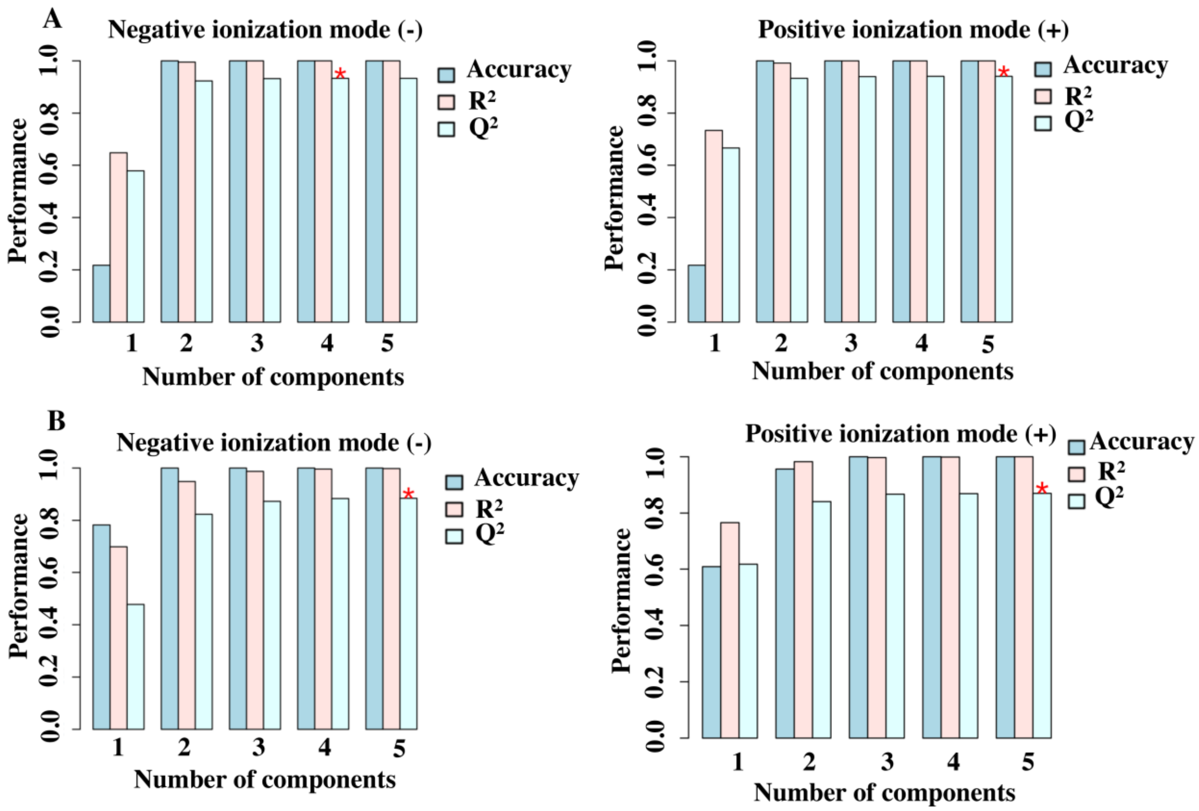

Figure S3. Cross validations of pLSDA of metabolomic (A) and lipidomic (B) analyses in negative and positive ionization modes.

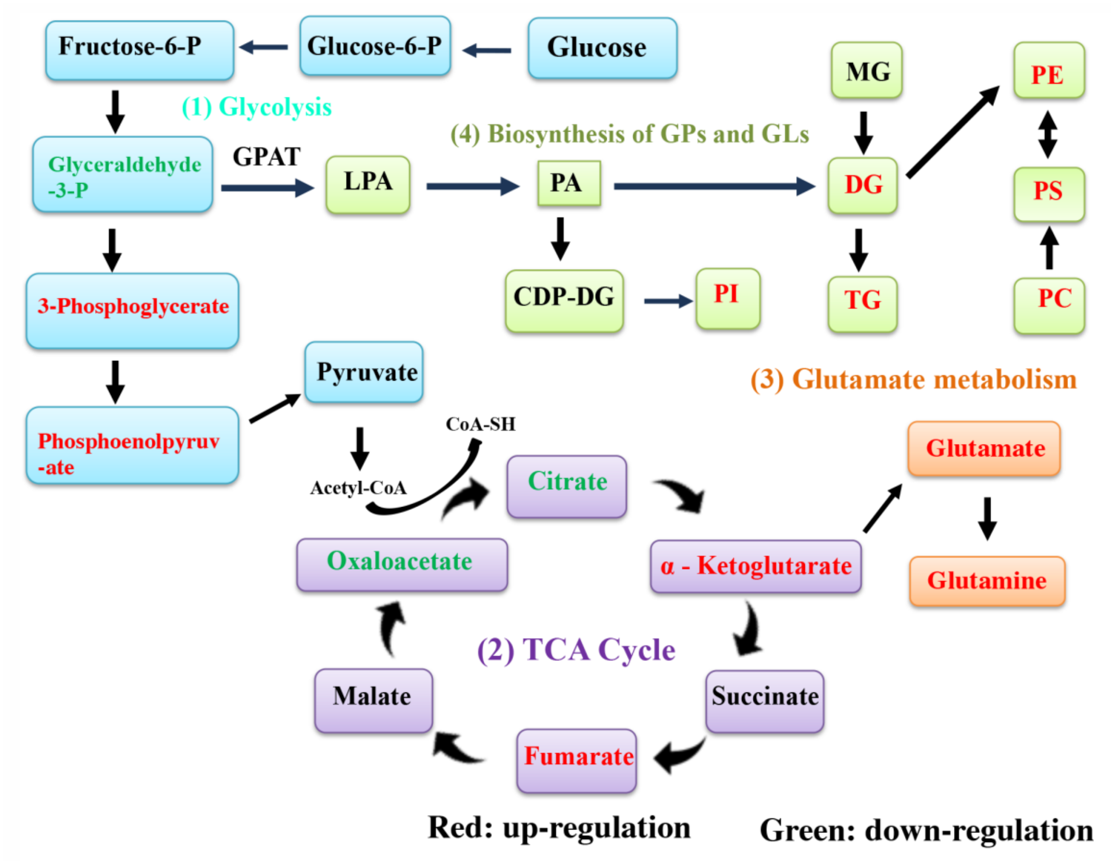

Figure S4. Metabolic and lipidomic networks describe the altered pathways in breast CCS after TCS exposure. Upregulated and downregulated metabolites and lipids were represented by red and green colors, respectively.

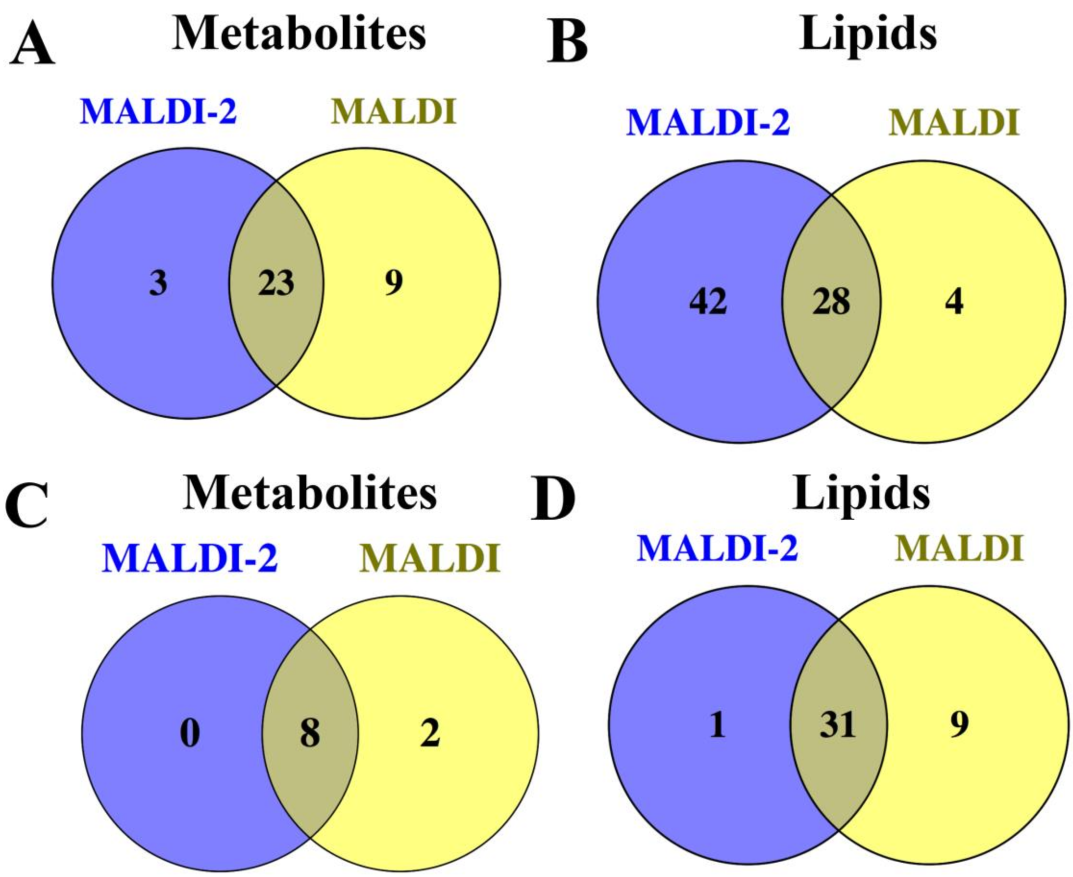

Figure S5. Venn diagrams for metabolites in positive (A) and negative (C) ionization modes. Venn diagrams for lipids in positive (B) and negative (D) ionization modes. Venn diagrams show shared metabolites and lipids between MALDI-2 and MALDI.

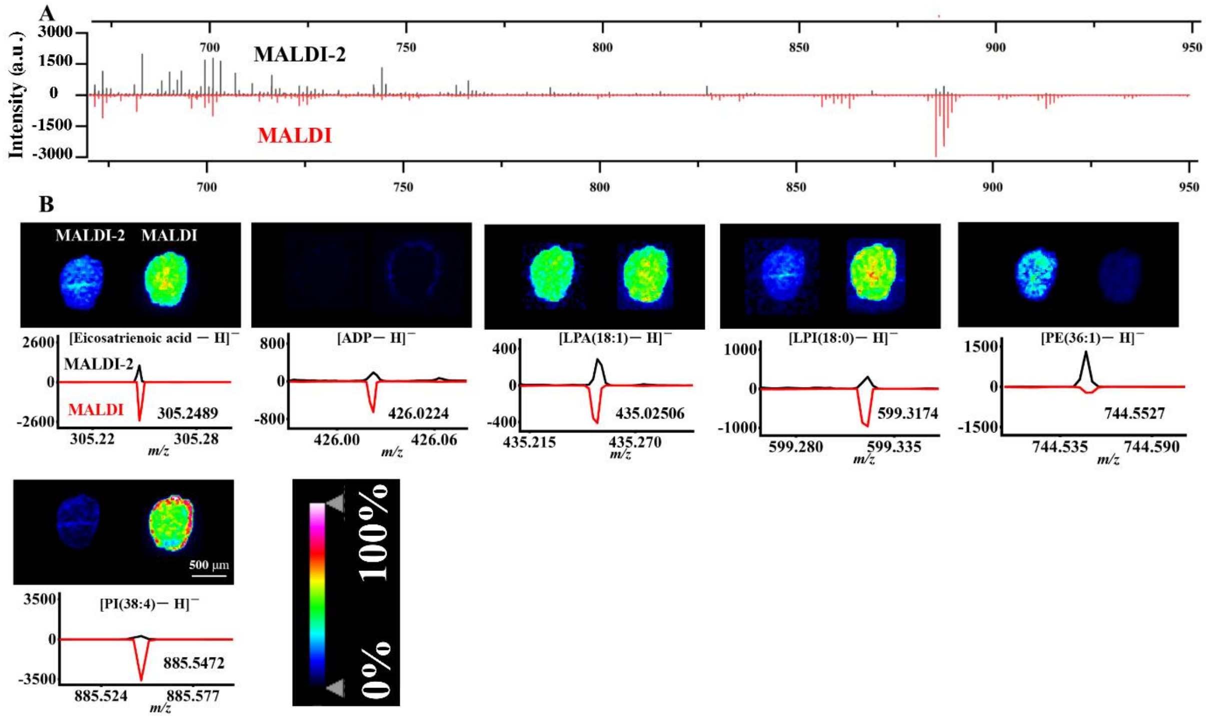

Figure S6. Mass spectra and ion images of MALD-2 and MALDI. (A) Mass spectra of MALDI-2 (black color) and MALDI (red color) in breast CCS sections in negative ionization mode using the 9AA matrix. (B) Representative MALDI-2 images (left) and MALDI images (right) of different ions in breast CCS sections. The corresponding ion spectra of MALDI-2 (upper black color) and MALDI (bottom red color) were listed below the ion images. The range of intensity values was indicated from 0 to 100%. The color gradient used was a heat map, with white indicating the highest intensity and blue indicating the lowest intensity. The intensity values were normalized to the TIC for each pixel.

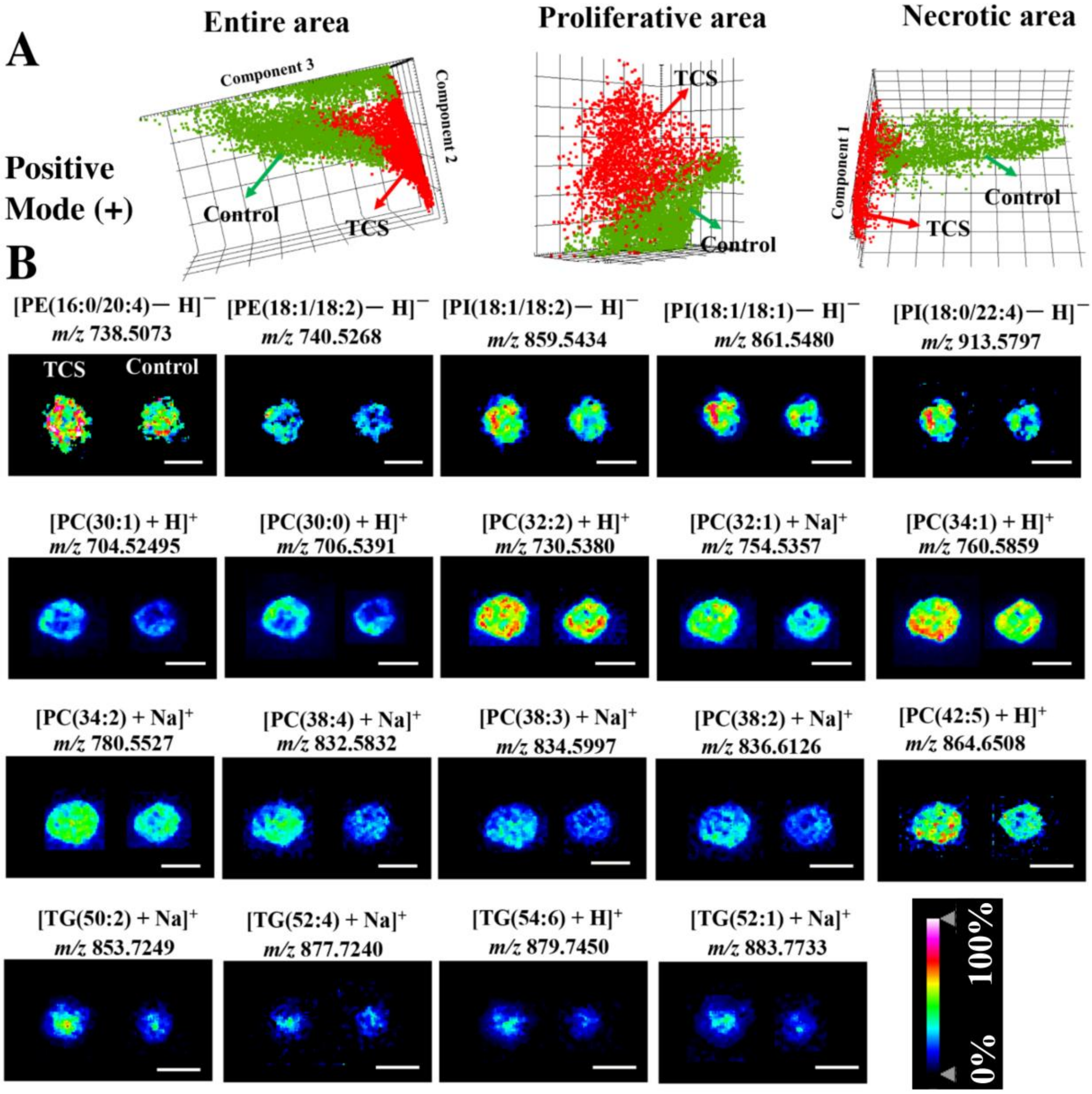

Figure S7. (A) pLSA score plots of MALDI-2 profiles in different CCS areas in positive ionization mode. (B) Representative ion images in positive and negative ionization modes. The range of intensity values was indicated from 0 to 100%. The color gradient used was a heat map, with white indicating the highest intensity and blue indicating the lowest intensity. The intensity values were normalized to the TIC for each pixel. All scale bars were 500 µm.

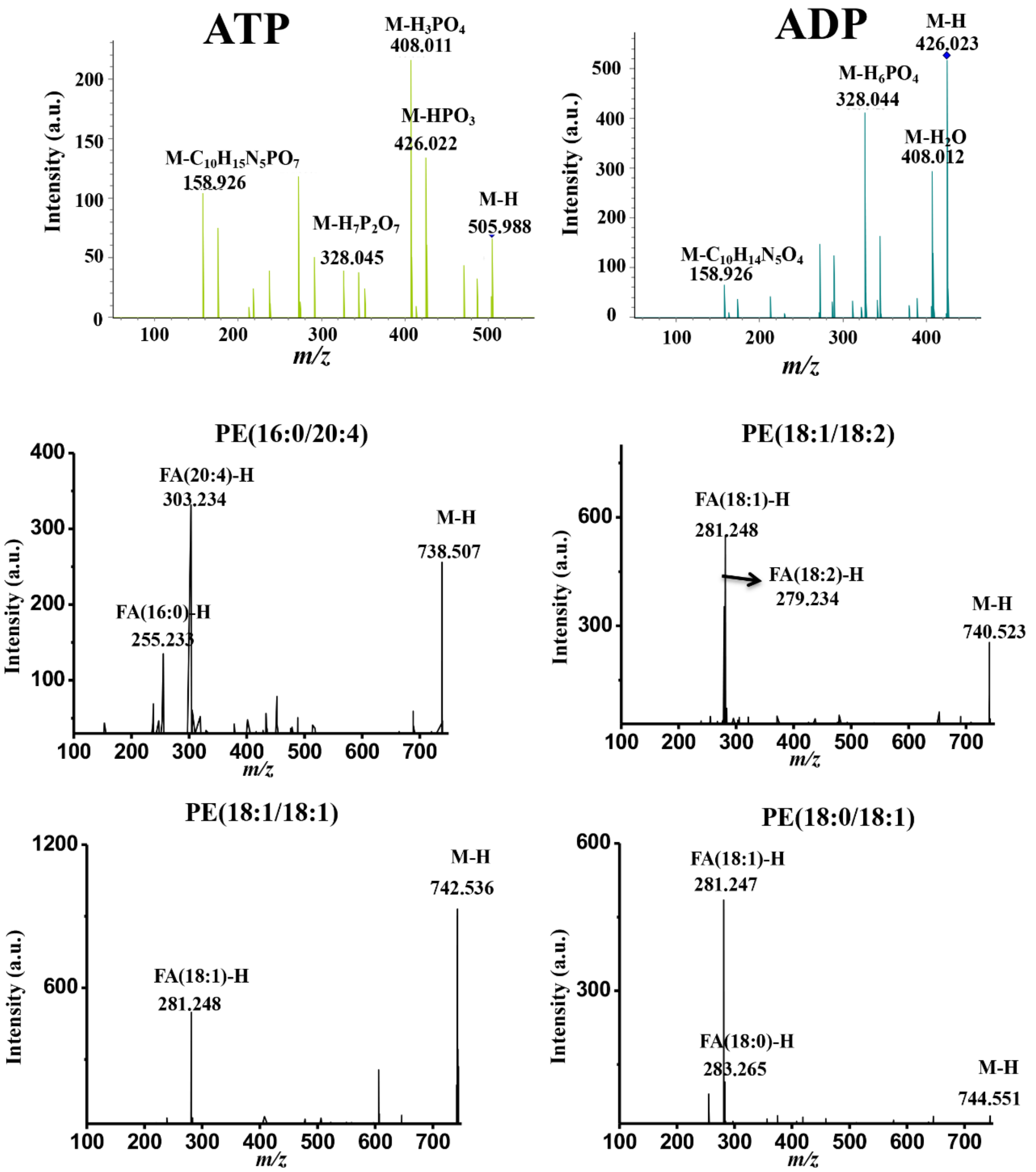
ATP
M-H_3PO_4
408.011
M-HPO_3
426.022
M-$C_{10}H_{15}N_5PO_7$
158.926
M-$H_7P_2O_7$
328.045
M-H
505.988
Intensity (a.u.)
m/z
ADP
M-H
426.023
M-H_6PO_4
328.044
M-H_2O
408.012
M-$C_{10}H_{14}N_5O_4$
158.926
PE(16:0/20:4)
FA(20:4)-H
303.234
M-H
738.507
FA(16:0)-H
255.233
PE(18:1/18:2)
FA(18:1)-H
281.248
FA(18:2)-H
279.234
M-H
740.523
PE(18:1/18:1)
M-H
742.536
FA(18:1)-H
281.248
PE(18:0/18:1)
FA(18:1)-H
281.247
FA(18:0)-H
283.265
M-H
744.551

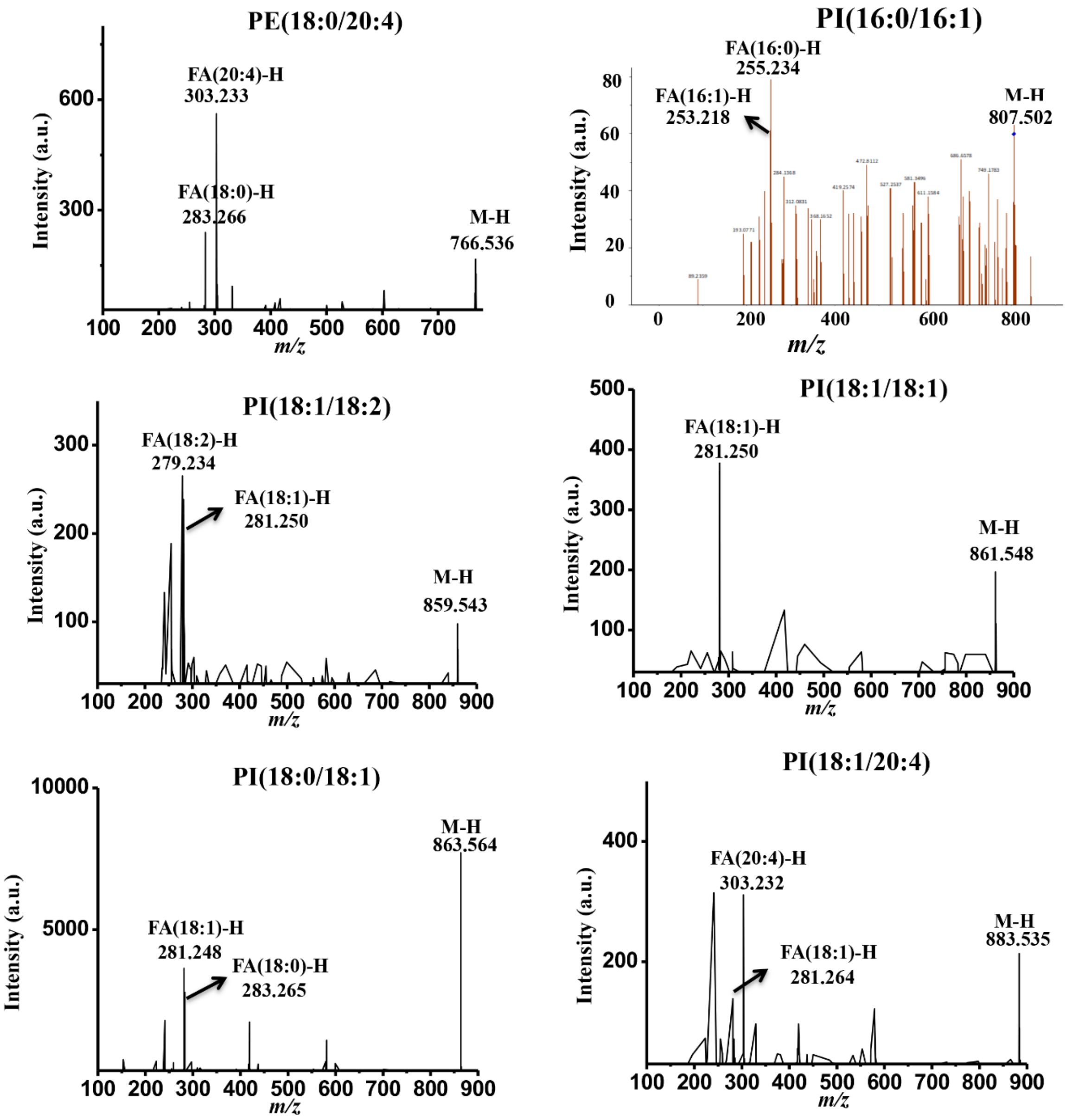
PE(18:0/20:4)
FA(20:4)-H
303.233
FA(18:0)-H
283.266
M-H
766.536
Intensity (a.u.)
m/z
PI(16:0/16:1)
FA(16:0)-H
255.234
FA(16:1)-H
253.218
M-H
807.502
Intensity (a.u.)
m/z
PI(18:1/18:2)
FA(18:2)-H
279.234
FA(18:1)-H
281.250
M-H
859.543
Intensity (a.u.)
m/z
PI(18:1/18:1)
FA(18:1)-H
281.250
M-H
861.548
Intensity (a.u.)
m/z
PI(18:0/18:1)
M-H
863.564
FA(18:1)-H
281.248
FA(18:0)-H
283.265
Intensity (a.u.)
m/z
PI(18:1/20:4)
FA(20:4)-H
303.232
FA(18:1)-H
281.264
M-H
883.535
Intensity (a.u.)
m/z

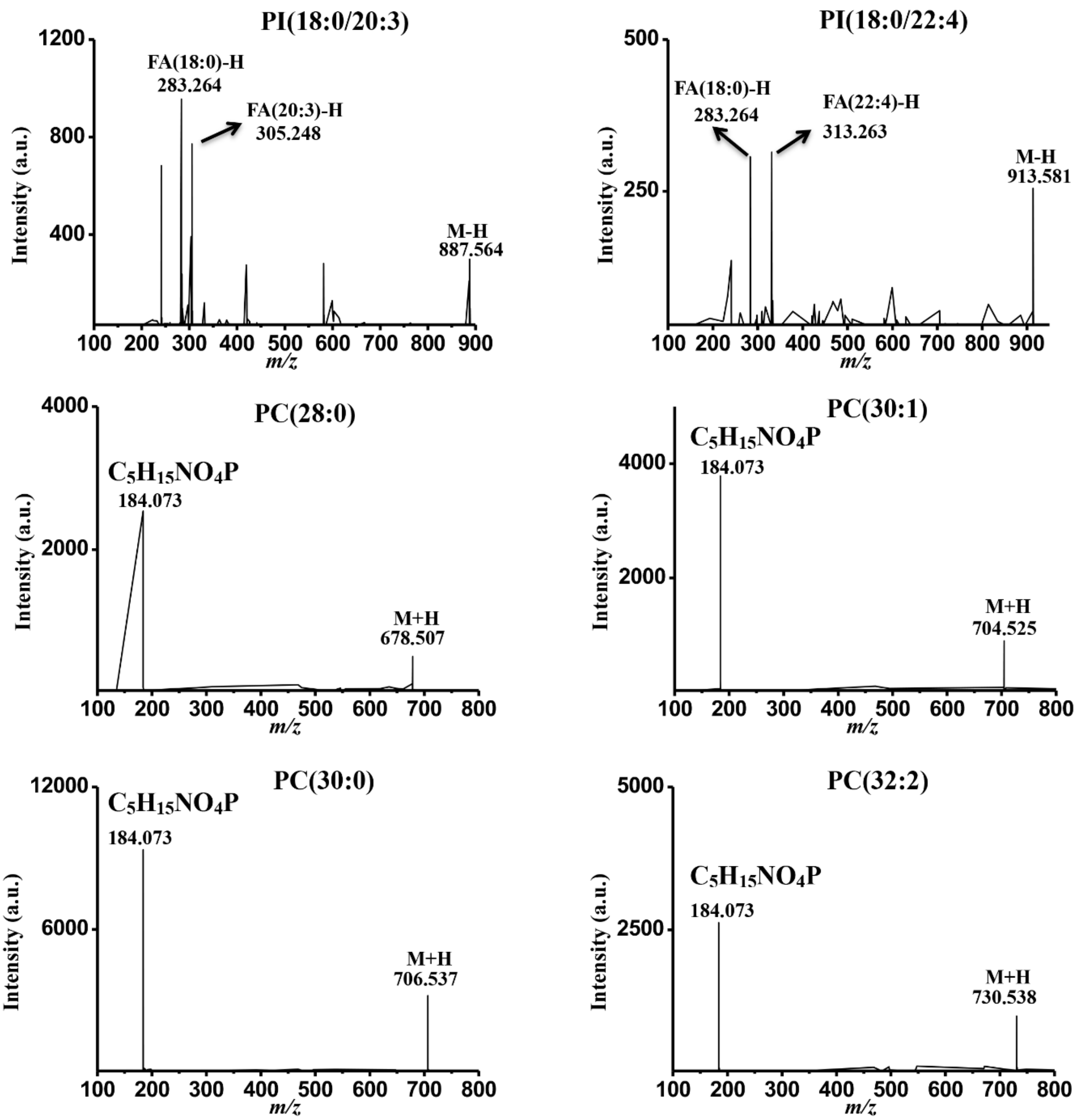
PI(18:0/20:3)
FA(18:0)-H
283.264
FA(20:3)-H
305.248
M-H
887.564
Intensity (a.u.)
m/z
PI(18:0/22:4)
FA(18:0)-H
283.264
FA(22:4)-H
313.263
M-H
913.581
PC(28:0)
C5H15NO4P
184.073
M+H
678.507
PC(30:1)
C5H15NO4P
184.073
M+H
704.525
PC(30:0)
C5H15NO4P
184.073
M+H
706.537
PC(32:2)
C5H15NO4P
184.073
M+H
730.538

108

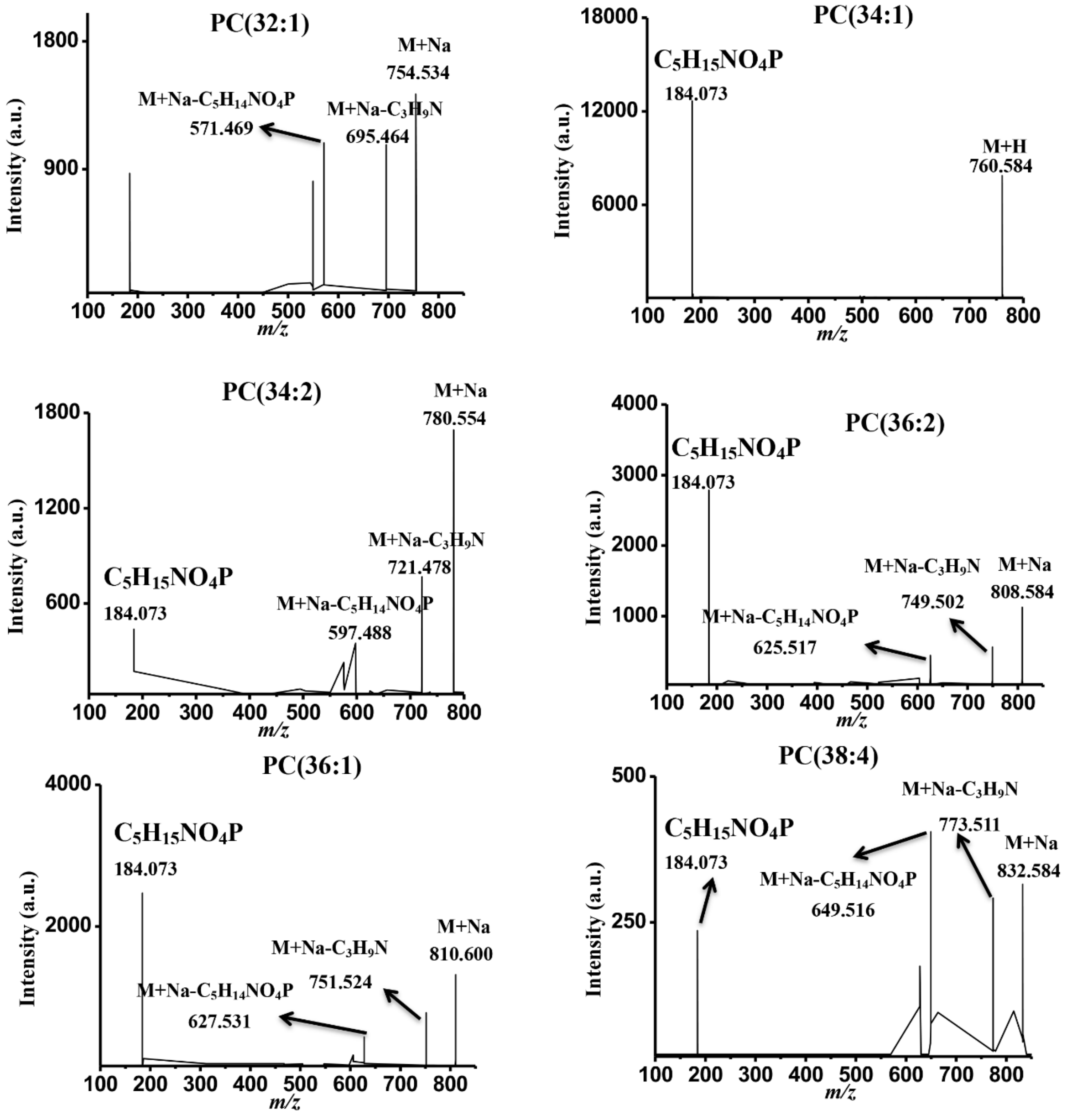
PC(32:1)
M+Na
754.534
M+Na-C5H14NO4P
571.469
M+Na-C3H9N
695.464
1800
900
Intensity (a.u.)
100 200 300 400 500 600 700 800
m/z
PC(34:1)
C5H15NO4P
184.073
M+H
760.584
18000
12000
6000
Intensity (a.u.)
100 200 300 400 500 600 700 800
m/z
PC(34:2)
M+Na
780.554
M+Na-C3H9N
721.478
C5H15NO4P
184.073
M+Na-C5H14NO4P
597.488
1800
1200
600
Intensity (a.u.)
100 200 300 400 500 600 700 800
m/z
PC(36:2)
C5H15NO4P
184.073
M+Na-C3H9N
749.502
M+Na
808.584
M+Na-C5H14NO4P
625.517
4000
3000
2000
1000
Intensity (a.u.)
100 200 300 400 500 600 700 800
m/z
PC(36:1)
C5H15NO4P
184.073
M+Na
810.600
M+Na-C3H9N
751.524
M+Na-C5H14NO4P
627.531
4000
2000
Intensity (a.u.)
100 200 300 400 500 600 700 800
m/z
PC(38:4)
M+Na-C3H9N
773.511
C5H15NO4P
184.073
M+Na
832.584
M+Na-C5H14NO4P
649.516
500
250
Intensity (a.u.)
100 200 300 400 500 600 700 800
m/z

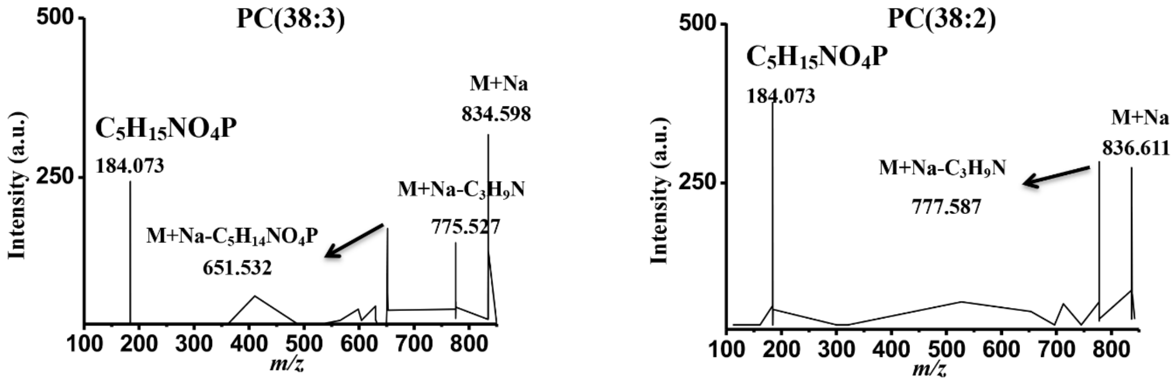

Figure S8. MALDI-MS/MS spectra of endogenous molecules in breast CCS.

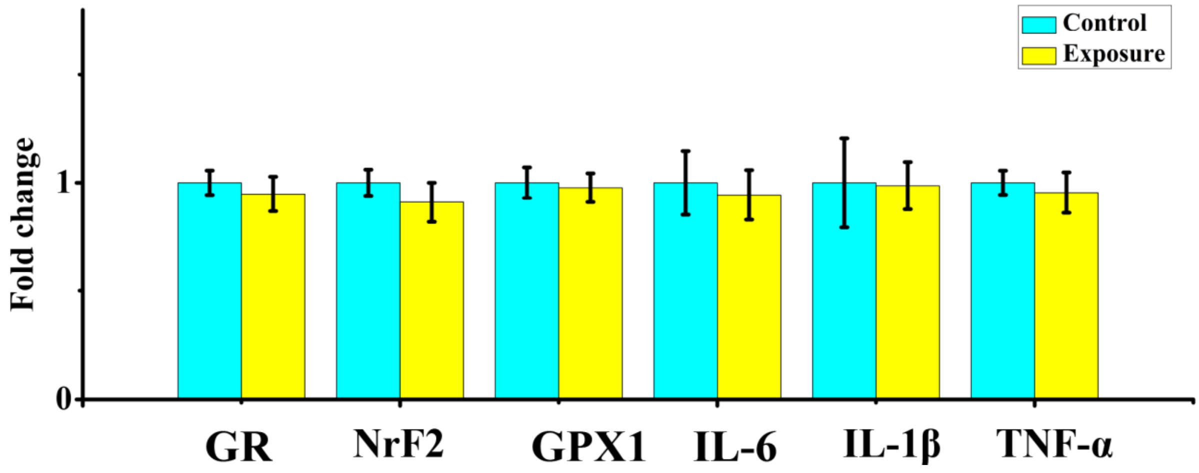

Figure S9. Fold changes of various genes related to reactive oxidative stress (GR, NrF2, GPX1) and inflammation (IL-6, IL-1β and TNF-α). (N =8)

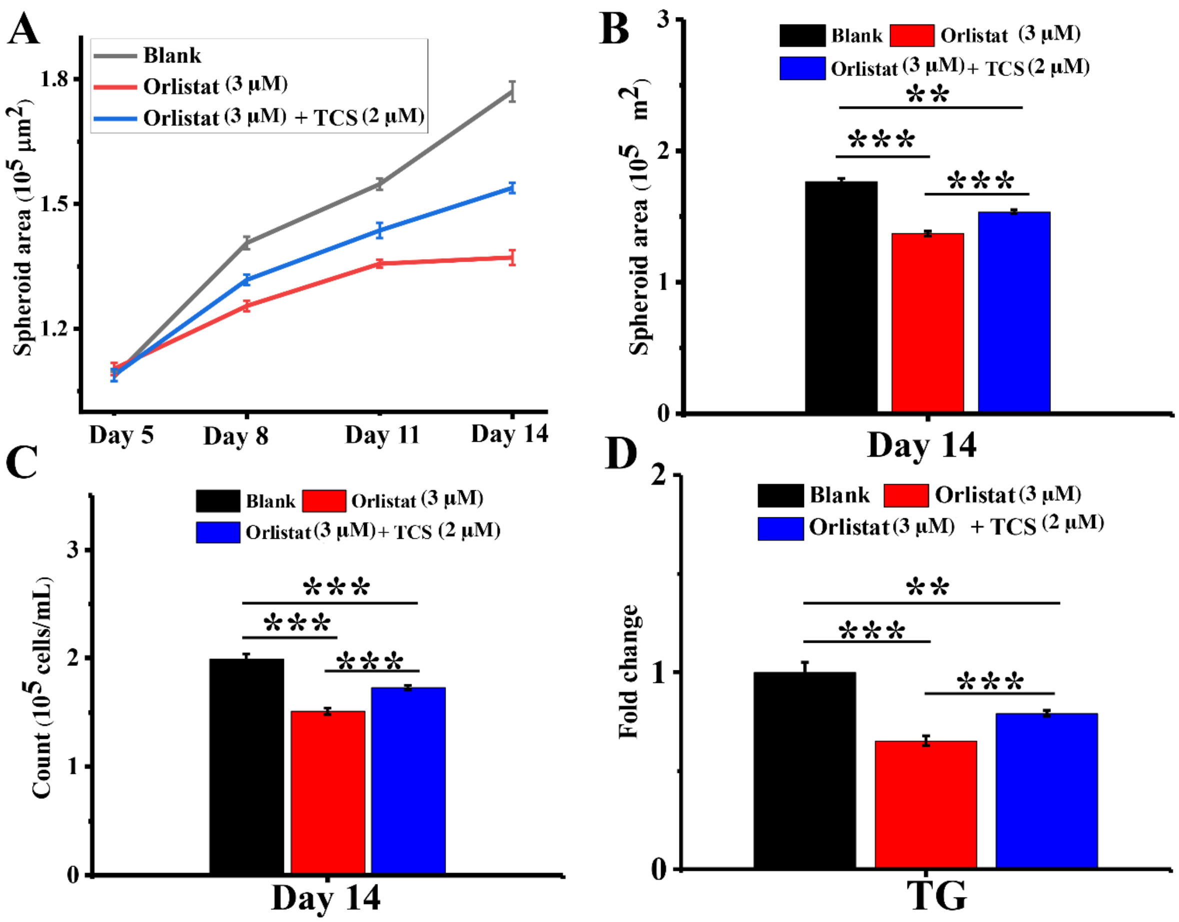

Figure S10. The effect of the accumulated triglyceride in MCF-7 breast CCS on their growth. (A) Growth curves of MCF-7 CCS exposed to orlistat (3 μM) or a combination of orlistat (3 μM) and TCS (2 μM) (n = 8). The areas (B) and the cell number (C) of breast CCS exposed to orlistat (3 μM) or a combination of orlistat (3 μM) and TCS (2 μM) (n = 8). (D) Fold changes of TG between control and exposure groups. (n = 6).

Table S1 The mobile phase, LC gradient and MS parameters for the analysis of metabolites by UPLC-MS/MS.

<table>
<tr><td>Mobile phase</td><td colspan="3">Positive ionization mode (Phase A: 95% ACN with 10 mM ammonium formate and 0.125% formic acid; Phase B: water with 0.125% formic acid and 10 mM ammonium formate)
Negative ionization mode (Phase A: 95% ACN with 10 mM ammonium acetate and 0.04% ammonium hydroxide; Phase B: water with 0.04% ammonium hydroxide and 10 mM ammonium acetate)</td></tr>
<tr><td rowspan="9">LC gradient</td><td>Flow Rate (mL/min)</td><td>Percentage B (%)</td><td>Time (min)</td></tr>
<tr><td>0.3</td><td>100</td><td>0.0</td></tr>
<tr><td>0.3</td><td>100</td><td>2.0</td></tr>
<tr><td>0.3</td><td>70</td><td>7.7</td></tr>
<tr><td>0.3</td><td>40</td><td>9.5</td></tr>
<tr><td>0.3</td><td>30</td><td>10.3</td></tr>
<tr><td>0.3</td><td>30</td><td>12.3</td></tr>
<tr><td>0.3</td><td>100</td><td>14.8</td></tr>
<tr><td>0.3</td><td>100</td><td>20.0</td></tr>
<tr><td>MS parameters</td><td colspan="3">Spray voltage:3.6 kV for positive ionization mode and 2.6 kV for negative ionization mode; Auxiliary gas heater temperature = 320 °C; Capillary temperature = 350 °C.Vaporizer temperature: 300 ℃; Sheath gas: 40 units;Auxiliary gas: 10 units; MS Resolution: 35 000; MS/MS resolution: 17 500; Full scan range:80-1200</td></tr>
</table>

Table S2 Instrumental method for the analysis of lipids by UPLC-MS/MS

Mobile phase	Phase A: ACN/H_2O (60:40, v/v) with 0.1% formic acid and 10 mM ammonium formate<br>Phase B: ACN/IPA (90:10, v/v) with 0.1% formic acid and 10 mM ammonium formate		
LC gradient	Flow Rate (mL/min)	Percentage B (%)	Time (min)
	0.26	70	0.0
	0.26	70	1.0
	0.26	55	2.0
	0.26	30	7.0
	0.26	15	9.0
	0.26	60	17.0
	0.26	60	19.0
	0.26	70	20.0
	0.26	70	23.0
MS parameters	Spray voltage:3.0 kV; Ion transfer tube temperature: 285 ℃; Vaporizer temperature: 300 ℃; Sheath gas: 50 units; Auxiliary gas: 15 units; Resolution: 35 000 for MS and 30 000 for MS^2; Scan range:100-1200		

Table S3 Instrumental methods for the quantitative analysis of TCS, TCSS and TCSG in culture medium and cancer cell spheroids by UPLC-MS/MS.

Analytical column	Hypersil Gold C18 column (1.7 µm, 2.1 mm × 100 mm, Thermo Fisher, MA, U.S.A.)		
Sampler temperature	4 ℃		
Column temperature	35 ℃		
Mobile phase	Phase A: 2.0 mM ammonium acetate aqueous solution<br>Phase B: methanol		
LC gradient	Flow Rate (mL/min)	Percentage B (%)	Time (min)
	0.3	85	0.0
	0.3	85	1.0
	0.3	20	2.5
	0.3	20	6.5
	0.3	85	7.0
	0.3	85	9.0
Injection volume	5 µL		

Table S4 Information of significantly changed metabolites detected by UPLC-MS/MS. The letter "N" and "P" mean the "negative" and "positive", respectively. Nine biological replicates were contained in each group.

No	Name	Retention time (min)	Detection mode	Fold change (Exp/Con, mean ± SD)	*p* value
1	ADP	10.36	N	1.40 ± 0.48	0.0433
2	ATP	10.53	N	1.32 ± 0.23	0.0104
3	Oxaloacetate	9.68	N	0.38 ± 0.35	0.0358
4	Alpha-Ketoglutarate	9.35	N	1.66 ± 0.46	0.0202
5	Fumarate	9.94	N	1.37 ± 0.21	0.0037
6	Citrate	10.24	N	0.55 ± 0.14	0.0139
7	Glyceraldehyde 3-phosphate	10.12	N	0.66 ± 0.07	0.0054
8	3-Phosphoglycerate	10.65	N	2.39 ± 0.55	<0.0001
9	Glycerol 3-phosphate	10.15	N	1.21 ± 0.20	0.0139
10	Glutamine	9.38	P	2.25 ± 0.78	0.0012
11	Glutamate	9.51	P	1.30 ± 0.24	0.0092
12	Tryptophan	7.78	P+N	1.79 ± 0.16	<0.0001
13	Phosphoenolpyruvic acid	10.32	N	1.39 ± 0.15	0.0093

14	Uridine diphosphate glucuronic acid	10.42	N	0.69 ± 0.07	<0.0001
15	Palmitoleic acid	1.37	N	1.54 ± 0.34	0.0013
16	N-Acetyl-L-methionine	6.66	N	1.99 ± 0.19	<0.0001
17	Galacturonic acid	9.70	N	3.04 ± 0.19	<0.0001
18	Beta-D-Glucopyranuronic acid(-)	9.95	N	1.75 ± 0.24	0.0004
19	Cytosine	7.71	P	1.91 ± 0.28	0.0007
20	Homoserine	9.11	P	1.89 ± 0.37	<0.0001
21	Taurine	8.67	P	1.25 ± 0.21	0.0210
22	Creatine	8.95	P	1.34 ± 0.25	0.0043
23	Guanine	7.43	P	1.31 ± 0.21	0.0035
24	Carnitine	8.07	P	0.64 ± 0.07	0.0376
25	Acetylspermidine	9.79	P	1.40 ± 0.36	0.0080
26	N6,N6,N6-Trimethyl-L-lysine	10.11	P	1.64 ± 0.26	<0.0001
27	Cytidine	7.68	P	1.52 ± 0.50	0.0139

Table S5 Information of significantly changed lipids analyzed by UPLC-MS/MS. The letter "N" and "P" mean the "negative" and "positive", respectively. For lipids detected in both negative and positive modes, their *p* values and fold changes in either negative mode or positive ionization modes were selected. For PI, PE, PS and PG, *p* values and fold changes in negative mode were selected. For PC, *p* values and fold changes in positive mode were selected. Nine biological replicates were contained in each group.

No	Name	Retention time (min)	Detection mode	Fold change (Exp/Con, mean ± SD)	*p* value
1	SM(d40:1)	10.68	P	0.57 ± 0.22	0.0247
2	SM(d39:0)	10.61	P	0.60 ± 0.20	0.0170
3	SM(d42:0)	11.07	P	0.60 ± 0.20	0.0036
4	SM(d41:0)	10.87	P	0.66 ± 0.16	0.0051
5	SM(d36:0)	9.88	P	0.71 ± 0.12	0.0439
6	SM(t34:1)	8.67	P	0.72 ± 0.11	0.0085
7	SM(t34:0)	8.50	P	0.75 ± 0.09	0.0403
8	SM(d41:1)	10.88	P	0.76 ± 0.09	0.0443
9	SM(d43:4)	10.88	P	0.76 ± 0.09	0.0454
10	SM(t34:2)	7.93	N	1.29 ± 0.29	0.0311
11	SM(d42:5)	9.36	P	1.57 ± 0.49	0.0351
12	SM(d36:5)	8.16	P	1.57 ± 0.49	0.0186
13	Cer(m18:0/22:0)	11.40	P	0.60 ± 0.34	0.0019

14	Cer(m18:0/23:0)	11.58	P	0.62 ± 0.32	0.0053
15	Cer(m18:0/24:0)	11.76	P	0.62 ± 0.32	0.0153
16	Cer(d18:1/22:0)	11.14	P	0.69 ± 0.28	0.0180
17	Cer(d18:1/24:0)	11.50	P	0.72 ± 0.25	0.0340
18	Cer(d18:0/24:1)	11.25	N	1.21 ± 0.09	0.0200
19	Cer(d17:1/16:0)	9.32	P	1.26 ± 0.13	0.0420
20	Cer(d18:1/16:0)	9.70	N	1.29 ± 0.15	0.0018
21	Cer(m18:1/16:0)	9.50	P	1.40 ± 0.23	0.0017
22	Cer(d18:2/16:0)	9.03	N	1.43 ± 0.25	0.0010
23	Cer(d18:2/24:1)	10.74	N	1.51 ± 0.31	0.0028
24	Cer(d16:1/24:1)	10.77	N	1.56 ± 0.34	0.0015
25	LPC(24:0)	7.78	P	1.56 ±0.00	0.0080
26	LPE(22:6)	2.19	N	1.43 ± 0.07	0.0455
27	LPE(18:1)	3.28	N	1.61 ± 0.07	0.0493
28	PC(16:0/22:4)	9.45	N	1.22 ± 0.33	0.0309
29	PC(16:0/20:3)	9.27	N	1.24 ± 0.32	0.0417
30	PC(16:0/22:6)	8.74	N	1.27 ± 0.30	0.0360
31	PC(16:0/16:1)	8.98	N	1.27 ± 0.30	0.0321
32	PC(18:0/22:3)	10.41	N	1.28 ± 0.29	0.0464
33	PC(16:0/22:5)	9.22	N	1.28 ± 0.29	0.0199
34	PC(36:1e)	10.48	P	1.29 ± 0.29	0.0480

35	PC(16:1/18:2)	8.41	N	1.29 ± 0.28	0.0465
36	PC(18:0/22:6)	9.43	N	1.29 ± 0.28	0.0127
37	PC(20:4/20:4)	8.21	N	1.31 ± 0.27	0.0179
38	PC(38:1)	10.60	P	1.31 ± 0.27	0.0252
39	PC(38:4e)	9.96	P	1.32 ± 0.26	0.0321
40	PC(16:0/18:2)	9.10	N	1.33 ± 0.26	0.0142
41	PC(22:2/18:2)	9.99	P	1.33 ± 0.26	0.0174
42	PC(42:8)	8.73	P	1.33 ± 0.26	0.0081
43	PC(16:1/20:4)	8.26	N	1.34 ± 0.25	0.0100
44	PC(30:0)	8.87	P	1.35 ± 0.24	0.0437
45	PC(18:1/18:1)	9.75	N	1.36 ± 0.23	0.0049
46	PC(18:1/22:0)	11.03	N	1.37 ± 0.23	0.0033
47	PC(40:6)	8.99	P	1.38 ± 0.22	0.0208
48	PC(22:0/20:4)	10.60	P	1.39 ± 0.22	0.0078
49	PC(38:3)	9.86	P	1.39 ± 0.21	0.0315
50	PC(14:1e/16:0)	9.23	P	1.39 ± 0.21	0.0252
51	PC(18:2/20:4)	8.39	N	1.42 ± 0.19	0.0045
52	PC(38:5e)	9.36	P	1.43 ± 0.18	0.0254
53	PC(38:6e)	9.24	P	1.44 ± 0.17	0.0339
54	PC(34:2)	9.07	P	1.46 ± 0.16	0.0209
55	PC(22:3/12:1)	8.16	P	1.47 ± 0.16	0.0159
56	PC(40:7)	8.74	P	1.48 ± 0.15	0.0031
57	PC(42:1)	11.35	P	1.49 ± 0.14	0.0091
58	PC(30:0e)	9.33	P	1.50 ± 0.14	0.0126

59	PC(42:10)	7.94	P	1.52 ± 0.12	0.0074
60	PC(34:3e)	9.39	P	1.52 ± 0.12	0.0088
61	PC(40:6)	8.99	P	1.53 ± 0.11	0.0124
62	PC(16:0e/16:0)	9.96	P	1.54 ± 0.11	0.0059
63	PC(28:0)	8.07	P	1.54 ± 0.11	0.0081
64	PC(34:1e)	9.97	P	1.56 ± 0.09	0.0074
65	PC(16:1e/18:1)	9.89	P	1.56 ± 0.09	0.0041
66	PC(38:4)	9.42	P	1.60 ± 0.06	0.0077
67	PC(32:0/12:4)	10.99	P	1.61 ± 0.06	0.0009
68	PC(36:3)	9.24	P	1.62 ± 0.05	0.0035
69	PC(38:7)	7.79	P	1.63 ± 0.05	0.0040
70	PC(30:1/10:0)	11.00	P	1.64 ± 0.04	0.0014
71	PC(18:1e/22:6)	9.14	P	1.64 ± 0.04	0.0055
72	PC(34:0)	10.12	P	1.65 ± 0.03	0.0407
73	PC(34:4)	8.15	P	1.66 ± 0.02	0.0207
74	PC(36:7)	8.15	P	1.66 ± 0.02	0.0207
75	PC(40:4)	10.00	P	1.67 ± 0.01	0.0010
76	PC(36:2e)	9.97	P	1.70 ± 0.01	0.0006
77	PC(42:9)	8.20	P	1.73 ± 0.02	0.0016
78	PC(42:2)	10.97	P	1.74 ± 0.03	0.0088
79	PC(38:6)	8.36	P	1.74 ± 0.03	0.0063
80	PC(44:5)	10.56	P	1.74 ± 0.04	0.0009
81	PC(40:2)	10.59	P	1.76 ± 0.05	0.0239
82	PC(36:2)	9.72	P	1.76 ± 0.05	0.0025

83	PC(36:6)	7.92	P	1.78 ± 0.07	0.0008
84	PC(14:0/14:0)	8.10	N	1.79 ± 0.07	0.0059
85	PC(34:5)	7.57	P	1.83 ± 0.10	0.0012
86	PC(32:2e)	8.74	P	1.84 ± 0.11	0.0113
87	PC(30:1/12:4)	10.11	P	1.85 ± 0.11	0.0258
88	PC(34:2e)	9.49	P	1.85 ± 0.12	0.0021
89	PC(20:3/20:4)	8.57	N	1.88 ± 0.13	<0.0001
90	PC(36:4)	8.94	P	1.88 ± 0.13	0.0109
91	PC(42:7)	9.05	P	1.89 ± 0.14	0.0257
92	PC(18:3e/18:0)	9.90	P	1.92 ± 0.16	0.0102
93	PC(36:5)	8.22	P	1.97 ± 0.19	0.0210
94	PC(36:4e)	9.35	P	2.01 ± 0.22	0.0012
95	PC(40:9)	7.62	P	2.12 ± 0.30	0.0001
96	PC(40:5)	9.44	P	2.19 ± 0.35	0.0008
97	PC(30:1)	8.14	P	2.22 ± 0.38	0.0005
98	PC(42:5)	9.91	P	2.23 ± 0.38	0.0177
99	PC(42:6)	9.48	P	2.23 ± 0.38	0.0003
100	PC(40:6e)	9.35	P	2.30 ± 0.43	0.0004
101	PC(32:2)	9.63	P	2.38 ± 0.49	0.0010
102	PC(16:1/22:5)	8.36	P	2.48 ± 0.56	0.0024
103	PC(38:5)	9.00	P	2.52 ± 0.58	0.0090
104	PC(34:1)	9.63	P	2.53 ± 0.59	0.0019
105	PC(20:4e/18:1)	9.36	P	2.61 ± 0.65	0.0009
106	PC(16:2e/16:0)	9.28	P	2.96 ± 0.90	0.0163

107	PC(40:5)	9.44	P	3.60 ± 1.35	0.0074
108	PE(14:0/14:0)	8.32	N	1.43 ± 0.11	0.0025
109	PE(14:0/18:2)	8.53	N	1.62 ± .03	<0.0001
110	PE(14:0/20:4)	8.38	P+N	1.44 ± 0.10	0.0011
111	PE(16:0/14:1)	8.58	P+N	1.89 ± 0.22	0.0076
112	PE(16:0/16:0)	9.75	P	1.47 ± 0.08	0.0287
113	PE(16:0/16:1)	9.16	N	1.36 ± 0.16	0.0102
114	PE(16:0/18:1)	9.12	P	1.98 ± 0.28	0.0003
115	PE(16:0/18:2)	9.29	N	1.47 ± 0.08	0.0003
116	PE(16:0/20:3)	9.44	N	1.53 ± 0.04	0.0001
117	PE(16:0/20:4)	9.15	P+N	1.26 ± 0.23	0.0331
118	PE(16:0/22:5)	9.40	N	1.45 ± 0.10	0.0005
119	PE(16:0/22:6)	8.92	P+N	1.46 ± 0.09	0.0087
120	PE(16:0p/18:1)	10.07	P	1.45 ± 0.09	0.0211
121	PE(16:0p/20:3)	9.72	P	1.67 ± 0.06	0.0027
122	PE(16:0p/22:6)	9.22	P	1.37 ± 0.15	0.0366
123	PE(16:1/14:0)	8.39	N	1.45 ± 0.09	0.0010
124	PE(16:1/18:2)	8.60	N	1.39 ± 0.14	0.0096
125	PE(16:1/20:3)	8.77	N	1.54 ± 0.03	0.0002
126	PE(16:1/20:4)	8.45	N	1.36 ± 0.16	0.0099
127	PE(16:1e/18:1)	10.10	N	1.27 ± 0.22	0.0396
128	PE(18:0/18:0)	10.01	P	1.91 ± 0.23	0.0006
129	PE(18:0/18:1)	9.77	P	1.51 ± 0.05	0.0038
130	PE(18:1/18:0)	10.34	N	1.33 ± 0.18	0.0107

131	PE(18:0/18:2)	9.83	N	1.42 ± 0.11	0.0021
132	PE(18:0/20:1)	10.76	N	1.30 ± 0.21	0.0168
133	PE(18:0/20:3)	10.04	P+N	1.50 ± 0.06	0.0005
134	PE(18:0/20:4)	9.77	P	1.77 ± 0.13	0.0012
135	PE(18:0/22:3)	10.54	N	1.42 ± 0.11	0.0029
136	PE(18:0/22:4)	10.17	P+N	1.24 ± 0.24	0.0428
137	PE(18:0/22:5)	9.79	N	1.32 ± 0.19	0.0125
138	PE(18:0/22:6)	9.59	P+N	1.44 ± 0.10	0.0012
139	PE(18:0p/18:1)	10.56	P	1.35 ± 0.17	0.0170
140	PE(18:0p/20:4)	10.04	P	1.31 ± 0.19	0.0475
141	PE(18:0p/22:5)	10.02	P	1.99 ± 0.29	0.0002
142	PE(18:1/18:1)	9.14	P	1.64 ± 0.04	0.0029
143	PE(18:1/18:2)	9.32	P+N	1.37 ± 0.15	0.0071
144	PE(18:1/20:3)	9.47	N	1.49 ± 0.07	0.0006
145	PE(18:1/22:5)	8.66	P	1.72 ± 0.10	0.0015
146	PE(18:1/22:6)	8.96	N	1.51 ± 0.05	0.0136
147	PE(18:1e/18:1)	10.59	N	1.29 ± 0.21	0.0199
148	PE(18:1e/22:5)	10.05	N	2.74 ± 0.82	0.0334
149	PE(18:1p/18:1)	10.07	P	1.46 ± 0.09	0.0352
150	PE(18:2e/18:1)	10.11	N	1.27 ± 0.23	0.0162
151	PE(20:1/18:1)	10.32	N	1.44 ± 0.11	0.0037
152	PE(22:5/20:4)	8.67	P	1.49 ± 0.07	0.0022
153	PE(28:0)	8.29	P	1.53 ± 0.04	0.0185
154	PE(32:1)	9.13	P	1.68 ± 0.06	0.0019

155	PE(34:1)	9.78	P	2.14 ± 0.39	0.0230
156	PE(34:2)	9.25	P	2.01 ± 0.30	0.0011
157	PE(34:2e)	10.07	P	1.62 ± 0.02	0.0357
158	PE(36:1)	10.31	P	1.40 ± 0.13	0.0065
159	PE(38:2)	9.98	P	1.80 ± 0.15	0.0008
160	PE(38:3)	10.01	P	1.91 ± 0.23	0.0006
161	PE(40:5)	9.76	P	1.62 ± 0.03	0.0030
162	PE(40:7)	8.93	P	3.00 ± 1.00	0.0000
163	PI(16:0/16:1)	8.36	N	1.39 ± 0.26	0.0207
164	PI(16:0/20:3)	8.70	N	2.33 ± 0.40	<0.0001
165	PI(18:0/18:1)	9.69	P+N	1.55 ± 0.15	0.0023
166	PI(18:0/20:3)	9.39	P+N	1.40 ± 0.26	0.0020
167	PI(18:0/22:6)	8.90	P+N	1.45 ± 0.22	0.0007
168	PI(18:1/18:1)	8.39	P	1.50 ± 0.19	0.0104
169	PI(18:1/18:2)	8.56	N	1.53 ± 0.17	0.0001
170	PI(18:1/20:3)	8.74	N	2.88 ± 0.79	0.0000
171	PI(18:1/20:4)	8.43	P+N	1.40 ± 0.26	0.0014
172	PI(34:1)	9.04	P	1.54 ± 0.15	0.0166
173	PI(36:2)	9.08	P	2.69 ± 0.66	0.0019
174	PI(40:4)	9.52	P	1.41 ± 0.25	0.0321
175	PS(18:0/18:0)	9.42	P	2.65 ± 0.03	0.0012
176	PS(18:0/18:1)	9.81	P+N	1.90 ± 0.50	0.0000
177	PS(18:0/20:3)	9.47	P+N	1.92 ± 0.49	0.0000
178	PS(18:0/20:4)	9.19	P+N	1.53 ± 0.76	0.0002

179	PS(18:0/22:4)	9.65	N	1.80 ± 0.57	0.0000
180	PS(18:0/22:5)	9.45	N	1.80 ± 0.57	0.0000
181	PS(18:0/22:6)	8.94	P	3.54 ± 0.66	0.0000
182	PS(36:2)	9.26	P	2.93 ± 0.23	0.0014
183	PS(40:4)	9.60	P	5.28 ± 1.89	0.0180
184	PS(40:5)	9.14	P	2.30 ± 0.22	0.0002
185	PG(16:0/14:0)	8.47	N	1.43 ± 0.15	0.0010
186	PG(16:0/16:0)	9.20	P+N	1.36 ± 0.20	0.0012
187	PG(16:0/16:1)	8.53	N	1.49 ± 0.11	0.0006
188	PG(16:0/18:1)	8.65	P	1.45 ± 0.13	0.0439
189	PG(16:0/18:2)	8.69	N	1.57 ± 0.05	0.0184
190	PG(16:1/20:4)	7.24	N	1.46 ± 0.13	0.0336
191	PG(18:0/18:1)	9.34	N	1.38 ± 0.19	0.0034
192	PG(18:1/18:1)	8.68	P	1.31 ± 0.24	0.0406
193	PG(18:1/18:2)	8.13	P	2.18 ± 0.38	0.0129
194	PG(18:1/22:5)	8.33	N	1.23 ± 0.29	0.0381
195	PG(18:1/22:6)	7.78	P	1.36 ± 0.20	0.0431
196	PG(18:2/20:4)	7.50	N	1.40 ± 0.17	0.0059
197	PG(18:3/20:4)	6.83	N	1.95 ± 0.22	0.0040
198	PG(20:0/18:1)	9.40	N	3.11 ± 1.03	0.0006
199	PG(20:1/20:4)	8.69	N	1.62 ± 0.02	0.0120
200	PG(20:3/18:2)	7.83	N	1.96 ± 0.23	0.0012
201	DG(12:0/20:4)	8.68	P	1.98 ± 0.18	0.0000
202	DG(12:0/22:5)	8.75	P	2.25 ± 0.01	0.0002

203	DG(14:0/20:3)	9.73	P	2.25 ± 0.01	0.0072
204	DG(14:0/20:4)	9.42	P	2.26 ± 0.02	0.0000
205	DG(14:0/22:6)	9.17	P	2.07 ± 0.12	0.0000
206	DG(15:0/16:0)	10.33	P	2.15 ± 0.06	0.0001
207	DG(15:0/16:1)	9.79	P	3.57 ± 0.9	0.0000
208	DG(15:0/18:1)	10.34	P	2.10 ± 0.10	0.0000
209	DG(16:0/14:0)	10.06	P	2.16 ± 0.06	0.0000
210	DG(16:0/16:0)	10.57	P	1.89 ± 0.24	0.0001
211	DG(16:0/16:1)	10.08	P	2.14 ± 0.07	0.0000
212	DG(16:0/17:0)	10.79	P	2.67 ± 0.30	0.0013
213	DG(16:0/18:1)	10.58	P	2.12 ± 0.08	0.0000
214	DG(16:0/20:3)	10.42	P	1.95 ± 0.20	0.0046
215	DG(16:0/20:4)	10.04	P	2.26 ± 0.02	0.0000
216	DG(16:0/22:0)	11.73	P	1.68 ± 0.39	0.0000
217	DG(16:0/22:6)	9.83	P	2.11 ± 0.09	0.0000
218	DG(16:0/24:1)	11.71	P	2.09 ± 0.10	0.0000
219	DG(16:1/12:0)	8.73	P	2.09 ± 0.10	0.0000
220	DG(16:1/14:0)	9.47	P	2.44 ± 0.15	0.0000
221	DG(16:1/14:1)	8.84	P	1.99 ± 0.17	0.0002
222	DG(16:1/16:1)	9.51	P	2.48 ± 0.17	0.0000
223	DG(16:1/18:1)	10.09	P	2.93 ± 0.49	0.0000
224	DG(16:1/20:4)	9.45	P	2.32 ± 0.06	0.0000
225	DG(16:1/22:5)	9.51	P	4.81 ± 1.82	0.0000
226	DG(16:1/22:6)	9.22	P	3.20 ± 0.68	0.0018

227	DG(17:0/18:1)	10.80	P	2.46 ± 0.16	0.0000
228	DG(17:0/20:3)	10.53	P	2.01 ± 0.16	0.0003
229	DG(17:0/20:4)	10.30	P	2.01 ± 0.16	0.0000
230	DG(17:1/16:1)	9.88	P	2.14 ± 0.07	0.0043
231	DG(17:1/18:1)	10.35	P	2.39 ± 0.11	0.0000
232	DG(17:1/18:2)	9.92	P	2.67 ± 0.31	0.0308
233	DG(17:1/20:4)	9.76	P	2.69 ± 0.32	0.0000
234	DG(18:0/16:0)	11.00	P	1.68 ± 0.39	0.0000
235	DG(18:0/18:0)	11.37	P	1.41 ± 0.58	0.0085
236	DG(18:0/18:1)	11.00	P	2.27 ± 0.03	0.0000
237	DG(18:0/20:1)	11.36	P	1.83 ± 0.29	0.0000
238	DG(18:0/20:3)	10.74	P	2.01 ± 0.16	0.0000
239	DG(18:0/20:4)	10.54	P	2.17 ± 0.05	0.0000
240	DG(18:0/22:3)	11.03	P	2.26 ± 0.01	0.0006
241	DG(18:0/22:4)	10.84	P	2.13 ± 0.08	0.0000
242	DG(18:0/22:5)	10.53	P	2.59 ± 0.25	0.0000
243	DG(18:0/22:6)	10.37	P	2.34 ± 0.08	0.0000
244	DG(18:1/14:1)	9.67	P	2.17 ± 0.04	0.0191
245	DG(18:1/18:1)	10.04	P	2.17 ± 0.05	0.0000
246	DG(18:1/18:2)	10.03	P	2.10 ± 0.09	0.0000
247	DG(18:1/20:3)	10.29	P	2.02 ± 0.15	0.0000
248	DG(18:1/20:4)	10.04	P	2.55 ± 0.22	0.0000
249	DG(18:1/22:1)	11.35	P	1.90 ± 0.24	0.0000
250	DG(18:1/22:4)	10.40	P	2.10 ± 0.10	0.0000

251	DG(18:1/22:5)	10.04	P	2.16 ± 0.05	0.0000
252	DG(18:1/22:6)	9.84	P	2.15 ± 0.06	0.0000
253	DG(18:1/24:0)	12.06	P	1.87 ± 0.26	0.0000
254	DG(18:1/24:1)	11.68	P	2.05 ± 0.13	0.0000
255	DG(18:2/14:1)	9.02	P	1.77 ± 0.33	0.0000
256	DG(19:0/18:1)	11.17	P	2.04 ± 0.14	0.0000
257	DG(19:1/18:1)	10.78	P	2.15 ± 0.06	0.0000
258	DG(19:1/20:3)	10.54	P	3.04 ± 0.57	0.0005
259	DG(19:1/20:4)	10.29	P	1.97 ± 0.19	0.0000
260	DG(20:1/22:5)	10.51	P	1.75 ± 0.34	0.0002
261	DG(20:1/22:6)	10.34	P	2.09 ± 0.10	0.0000
262	DG(20:3/20:4)	9.69	P	2.80 ± 0.40	0.0000
263	DG(20:3/22:4)	10.08	P	2.23 ± 0.00	0.0001
264	DG(20:4/20:4)	9.37	P	3.85 ± 1.14	0.0000
265	DG(22:0/20:4)	11.15	P	1.83 ± 0.29	0.0000
266	DG(22:1/20:4)	10.82	P	2.21 ± 0.01	0.0004
267	DG(22:4/20:4)	9.82	P	1.94 ± 0.21	0.0001
268	DG(22:5/20:4)	9.39	P	2.37 ± 0.10	0.0001
269	DG(22:6/14:1)	8.65	P	2.24 ± 0.01	0.0000
270	DG(24:0/20:4)	11.68	P	2.12 ± 0.08	0.0000
271	DG(24:1/20:4)	11.29	P	2.12 ± 0.08	0.0000
272	DG(30:0e)	10.07	P	2.36 ± 0.09	0.0000
273	DG(30:1e)	12.43	P	2.05 ± 0.13	0.0286
274	DG(32:2e)	9.15	P	2.08 ± 0.11	0.0000

275	DG(34:1e)	10.58	P	2.06 ± 0.13	0.0000
276	DG(34:2e)	8.67	P	1.76 ± 0.33	0.0000
277	DG(36:3e)	9.69	P	2.04 ± 0.14	0.0000
278	DG(36:4e)	9.21	P	1.75 ± 0.34	0.0004
279	DG(38:5e)	10.49	P	1.84 ± 0.28	0.0000
280	DG(39:4)	10.75	P	2.05 ± 0.13	0.0000
281	MG(20:3)	4.20	P	1.28 ± 0.06	0.0370
282	MG(22:4)	4.58	P	1.45 ± 0.06	0.0431
283	TG(11:0/14:0/16:0)	11.91	P	2.35 ± 0.36	0.0020
284	TG(12:0/14:0/14:0)	11.74	P	1.77 ± 0.05	0.0000
285	TG(12:1e/6:0/20:4)	10.04	P	2.37 ± 0.38	0.0000
286	TG(14:0/14:0/14:1)	11.76	P	1.70 ± 0.10	0.0123
287	TG(15:0/14:0/14:0)	12.25	P	1.51 ± 0.23	0.0002
288	TG(15:0/16:0/16:0)	12.89	P	1.40 ± 0.31	0.0174
289	TG(15:0/16:0/18:1)	12.95	P	1.28 ± 0.40	0.0109
290	TG(15:0/16:0/18:3)	12.27	P	3.04 ± 0.85	0.0002
291	TG(15:0/16:1/16:1)	12.27	P	1.56 ± 0.20	0.0000
292	TG(15:0/16:1/18:1)	12.59	P	1.47 ± 0.26	0.0008
293	TG(16:0/12:0/14:0)	12.09	P	1.42 ± 0.29	0.0001
294	TG(16:0/14:0/14:0)	12.44	P	1.32 ± 0.37	0.0006
295	TG(16:0/14:0/17:1)	12.59	P	1.37 ± 0.33	0.0036
296	TG(16:0/14:0/20:4)	12.34	P	1.76 ± 0.05	0.0002
297	TG(16:0/14:0/22:6)	12.18	P	1.41 ± 0.30	0.0001
298	TG(16:0/14:1/14:1)	11.79	P	1.82 ± 0.01	0.0000

299	TG(16:0/14:1/16:0)	12.43	P	1.69 ± 0.11	0.0067
300	TG(16:0/14:1/20:4)	12.01	P	1.51 ± 0.23	0.0192
301	TG(16:0/14:1/22:6)	11.85	P	1.72 ± 0.08	0.0003
302	TG(16:0/16:0/18:1)	13.13	P	2.05 ± 0.15	0.0005
303	TG(16:0/16:0/18:3)	12.43	P	2.80 ± 0.68	0.0001
304	TG(16:0/16:0/22:6)	12.52	P	1.51 ± 0.23	0.0000
305	TG(16:0/16:1/22:6)	12.18	P	1.56 ± 0.20	0.0222
306	TG(16:0/17:0/20:4)	12.86	P	2.89 ± 0.75	0.0022
307	TG(16:0/17:1/18:1)	12.94	P	1.32 ± 0.37	0.0063
308	TG(16:0/17:1/20:4)	12.49	P	2.00 ± 0.11	0.0090
309	TG(16:0/17:1/22:6)	12.34	P	2.23 ± 0.28	0.0169
310	TG(16:0/18:1/23:1)	13.97	P	1.74 ± 0.07	0.0252
311	TG(16:0/20:4/22:6)	12.10	P	1.29 ± 0.39	0.0017
312	TG(16:0e/14:0/16:0)	13.31	P	1.46 ± 0.27	0.0005
313	TG(16:0e/18:0/22:6)	13.37	P	1.77 ± 0.05	0.0202
314	TG(16:0e/18:1/22:4)	13.43	P	0.35 ± 1.05	0.0420
315	TG(16:0e/18:1/22:6)	12.99	P	1.47 ± 0.26	0.0023
316	TG(16:1/14:0/14:0)	12.09	P	1.67 ± 0.12	0.0001
317	TG(16:1/14:0/16:1)	12.11	P	1.57 ± 0.19	0.0029
318	TG(16:1/14:0/18:1)	12.43	P	2.18 ± 0.25	0.0006
319	TG(16:1/14:0/18:3)	11.85	P	1.49 ± 0.25	0.0485
320	TG(16:1/14:1/16:1)	11.82	P	1.65 ± 0.14	0.0001
321	TG(16:1/14:1/17:1)	11.98	P	1.69 ± 0.10	0.0333
322	TG(16:1/14:1/20:4)	11.71	P	3.49 ± 1.17	0.0000

323	TG(16:1/16:1/16:1)	12.13	P	1.53 ± 0.22	0.0000
324	TG(16:1/17:1/18:1)	12.60	P	1.45 ± 0.28	0.0013
325	TG(16:1/17:1/20:4)	12.17	P	1.53 ± 0.22	0.0000
326	TG(16:1e/14:0/16:0)	12.92	P	1.76 ± 0.05	0.0007
327	TG(16:1e/16:0/16:1)	12.92	P	2.03 ± 0.14	0.0000
328	TG(17:0/20:4/20:4)	12.34	P	1.93 ± 0.07	0.0485
329	TG(18:0/16:0/20:4)	13.04	P	1.33 ± 0.36	0.0021
330	TG(18:0/16:0/22:6)	12.87	P	1.58 ± 0.18	0.0057
331	TG(18:0/17:1/18:1)	13.29	P	1.37 ± 0.33	0.0010
332	TG(18:0/18:0/20:4)	13.39	P	1.96 ± 0.08	0.0343
333	TG(18:0/18:0/22:4)	13.63	P	1.71 ± 0.09	0.0322
334	TG(18:0/18:0/22:6)	13.21	P	2.56 ± 0.51	0.0004
335	TG(18:0/18:1/18:1)	13.46	P	1.56 ± 0.19	0.0003
336	TG(18:0/18:1/22:5)	12.97	P	1.84 ± 0.00	0.0040
337	TG(18:0/18:1/22:6)	12.86	P	1.83 ± 0.01	0.0019
338	TG(18:0/20:1/22:4)	13.69	P	2.92 ± 0.76	0.0076
339	TG(18:0/20:4/22:4)	12.82	P	2.70 ± 0.61	0.0090
340	TG(18:0/20:4/22:6)	12.42	P	3.35 ± 1.07	0.0053
341	TG(18:0e/16:1/16:1)	13.27	P	1.51 ± 0.24	0.0004
342	TG(18:1/14:0/18:3)	12.16	P	1.67 ± 0.12	0.0076
343	TG(18:1/17:1/20:4)	12.48	P	2.73 ± 0.63	0.0000
344	TG(18:1/18:1/18:2)	12.83	P	2.89 ± 0.74	0.0129
345	TG(18:1/18:1/18:3)	12.67	P	1.44 ± 0.28	0.0013
346	TG(18:1/18:1/20:4)	12.64	P	1.98 ± 0.10	0.0000

347	TG(18:1/18:1/22:0)	14.15	P	1.29 ± 0.38	0.0042
348	TG(18:1/18:1/22:1)	13.78	P	1.80 ± 0.03	0.0040
349	TG(18:1/18:1/22:5)	12.61	P	1.64 ± 0.14	0.0006
350	TG(18:1/18:1/22:6)	12.49	P	2.63 ± 0.56	0.0307
351	TG(18:1/18:1/24:1)	14.13	P	1.33 0.36	0.0028
352	TG(18:1/20:4/22:5)	12.21	P	1.94 ± 0.07	0.0174
353	TG(18:1/20:4/22:6)	12.08	P	1.43 ± 0.29	0.0009
354	TG(18:1e/14:0/16:0)	13.29	P	1.47 ± 0.26	0.0005
355	TG(18:1e/16:0/18:1)	13.63	P	1.42 ± 0.30	0.0003
356	TG(19:1/18:0/20:3)	13.38	P	2.29 ± 0.32	0.0249
357	TG(19:1/18:1/18:1)	13.27	P	2.67 ± 0.59	0.0339
358	TG(20:4/14:1/20:4)	11.65	P	1.63 ± 0.15	0.0000
359	TG(26:0/18:1/18:1)	14.89	P	1.58 ± 0.18	0.0022
360	TG(4:0/16:0/18:1)	11.19	P	2.11 ± 0.19	0.0000
361	TG(4:0/16:1/16:1)	10.44	P	2.27 ± 0.31	0.0000
362	TG(4:0_18:1_18:1)	11.16	P	1.74 ± 0.07	0.0080

Table S6 Information of endogenous metabolites and lipids in positive ionization mode detected by MALDI and MALDI-2 MSI. Symbols, “+” and “-”, meant the meaningful and unmeaningful spatial distributions of endogenous molecules in breast CCS, respectively. Fold changes were the average intensities of metabolites in M2 divided by those in M1. Five biological replicates were contained in each group.

No	Name	Ion adduct	Theoretical *m/z*	Experimental *m/z*	|Δ ppm|	M2	M1	Fold change (M2/M1)
1	2-Hexenoylcholine	M+Na	223.1542	223.1543	0.32	+	+	0.31
2	Spermine	M+Na	225.2043	225.2050	2.94	+	+	0.77
3	Tetradecanol	M+Na	237.2188	237.2189	0.33	+	+	0.36
4	Ecgonine methyl ester	M+K	238.0839	238.0840	0.45	-	+	
5	N-a-Acetyl-L-arginine	M+Na	239.1122	239.1115	3.11	+	+	0.20
6	Propionylcarnitine	M+Na	240.1198	240.1206	3.44	+	+	0.56
7	1-Hexadecanol	M+Na	265.2500	265.2502	0.71	+	+	0.86
8	Glutaconylcarnitine	M+H	274.1287	274.1285	0.65	+	-	
9	Oleamide	M+H	282.2781	282.2791	3.64	+	+	0.22
10	3-Oxooctadecanoic acid	M+H	299.2569	299.2581	3.91	-	+	
11	Atenolol	M+K	305.1268	305.1262	1.92	+	-	
12	Phytosphingosine	M+H	318.2994	318.3003	2.75	-	+	
13	dUMP	M+Na	331.0294	331.0302	2.34	+	+	0.18
14	Phenylalanylphenylalanine	M+Na	335.1379	335.1366	3.83	+	+	1.93
15	Pregnanetriol	M+H	337.2733	337.2733	0.10	+	+	1.22
16	Dolichol phosphate	M+K	343.1429	343.1435	1.80	+	+	17.19

17	Tetradecanoylcarnitine	M+H	372.3100	372.3108	2.24	+	+	0.28
18	2-Arachidonylglycerol	M+H	379.2827	379.2843	4.18	+	+	4.87
19	L-Palmitoylcarnitine	M+H	400.3410	400.3421	2.86	+	+	2.00
20	2-Hydroxymyristoylcarnitine	M+Na	410.2869	410.2877	1.90	-	+	
21	Dodecanedioylcarnitine	M+K	412.2095	412.2096	0.21	+	+	0.50
22	Heptadecanoyl carnitine	M+H	414.3565	414.3578	3.06	-	+	
23	Hydroxyhexadecanoyl carnitine	M+H	416.3377	416.3370	1.56	-	+	
24	Stearoylcarnitine	M+H	428.3740	428.3734	1.34	+	+	0.41
25	Tetrahydrofolic acid	M+H	446.1796	446.1783	2.99	+	-	
26	Arachidyl carnitine	M+H	456.4054	456.4047	1.45	-	+	
27	3-Sulfodeoxycholic acid	M+H	459.2416	459.2411	1.08	+	+	0.45
28	Leukotriene E4	M+Na	462.2271	462.2285	2.98	+	+	2.11
29	Trihydroxycoprostanoic acid	M+H	465.3577	465.3575	0.50	-	+	
30	Trihydroxycoprostanoic acid	M+Na	487.3372	487.3394	4.53	-	+	
31	Citicoline	M+H	489.1143	489.1146	0.63	+	+	3.43
32	Cervonyl carnitine	M+Na	494.3240	494.3241	0.18	+	+	0.43
33	LPC 16:0	M+H	496.3421	496.3398	4.63	+	+	0.73
34	Tauroursodeoxycholic acid	M+H	500.3041	500.3040	0.14	+	+	3.37

35	LPC 18:1	M+H	522.3560	522.3554	1.14	+	+	0.71
36	Hexacosanoyl carnitine	M+H	540.4980	540.4986	1.13	-	+	
37	DG O-32:2	M+H	551.5049	551.5034	2.72	+	+	3.96
38	PC 22:1	M+H	592.3999	592.3973	4.39	+	+	1.12
39	DG O-36:4	M+H	603.5347	603.5347	0.00	+	+	3.73
40	DG 36:4	M+H	617.5159	617.5139	3.24	+	-	
41	LPI 20:1	M+H	627.3476	627.3504	4.46	+	-	
42	LPI 22:2	M+H	653.3638	653.3660	3.37	+	-	
43	PA 34:4	M+H	669.4474	669.4490	2.39	+	-	
44	PC 28:0	M+H	678.5072	678.5068	0.59	+	+	0.48
45	PA 36:5	M+H	695.4650	695.4646	0.58	+	-	
46	PA 36:4	M+H	697.4798	697.4803	0.72	+	-	
47	SM 34:1	M+H	703.5763	703.5748	2.13	+	+	0.55
48	PC 30:1	M+H	704.5249	704.5225	3.41	+	+	0.49
49	SM 34:0	M+H	705.5897	705.5905	1.13	+	+	0.64
50	PC 30:0	M+H	706.5391	706.5381	1.42	+	+	0.65
51	PE 34:1	M+H	718.5371	718.5381	1.39	+	+	1.57
52	PA 38:6	M+H	721.4786	721.4803	2.36	+	-	
53	PA 38:5	M+H	723.4928	723.4959	4.28	+	-	
54	SM 34:1	M+Na	725.5574	725.5568	0.83	+	+	2.06
55	PC 30:0	M+Na	728.5199	728.5201	0.27	-	+	
56	PC 32:2	M+H	730.5379	730.5381	0.27	+	+	0.54
57	PC 32:1	M+H	732.5529	732.5538	1.23	+	+	0.52

58	PC 32:0	M+H	734.5695	734.5694	0.14	+	+	0.66
59	PE 36:1	M+H	746.5717	746.5694	3.08	+	+	1.50
60	PE 36:0	M+H	748.5858	748.5851	0.94	+	+	1.30
61	PI 28:2	M+H	751.4388	751.4392	0.53	+	-	
62	PA 40:5	M+H	751.5264	751.5272	1.06	+	-	
63	PC 32:1	M+Na	754.5357	754.5357	0.00	+	+	1.68
64	PC 34:3	M+H	756.5512	756.5538	3.44	+	+	1.35
65	PC 34:2	M+H	758.5700	758.5694	0.79	+	+	0.56
66	PC 34:1	M+H	760.5859	760.5851	1.05	+	+	0.76
67	PS 34:1	M+H	762.5259	762.5280	2.75	+	-	
68	PS O-36:5	M+H	768.5195	768.5174	2.73	+	-	
69	PE 38:4	M+H	768.5575	768.5538	4.81	+	-	
70	PE 38:2	M+H	772.5841	772.5851	1.29	+	+	2.01
71	PE 38:1	M+H	774.6020	774.6007	1.68	+	+	2.28
72	PC 34:2	M+Na	780.5527	780.5514	1.67	-	+	
73	PC 34:1	M+Na	782.5676	782.5670	0.77	-	+	
74	PC 34:0	M+Na	784.5809	784.5827	2.29	+	+	0.89
75	PC 36:2	M+H	786.6019	786.6007	1.53	+	+	0.52
76	PC 36:1	M+H	788.6153	788.6164	1.39	+	+	0.63
77	PS 36:0	M+H	792.5734	792.5749	1.89	+	-	
78	Thyroxine	M+Na	799.6729	799.6759	3.78	+	+	3.86
79	PC 36:2	M+Na	808.5844	808.5827	2.10	+	+	1.22
80	PC 36:1	M+Na	810.5993	810.5983	1.23	+	+	
81	PG 36:2	M+K	813.5023	813.5042	2.34	+	-	

82	SM 42:2	M+H	813.6828	813.6844	1.97	+	+	0.76
83	PC 38:2	M+H	814.6284	814.6320	4.42	-	+	
84	SM 42:1	M+H	815.7025	815.7000	3.06	+	+	0.78
85	SM 42:0	M+H	817.7164	817.7157	0.86	+	+	0.70
86	PI O-34:3	M+H	819.5363	819.5382	2.32	+	-	
87	TG 48:1	M+Na	827.7079	827.7099	2.42	+	-	
88	PC 38:4	M+Na	832.5832	832.5827	0.60	+	-	
89	PI 34:3	M+H	833.5142	833.5175	3.96	+	-	
90	Coenzyme Q9	M+K	833.5868	833.5845	2.76	+	+	1.92
91	PC 38:3	M+Na	834.5997	834.5983	1.68	-	+	
92	PC 38:2	M+Na	836.6126	836.6140	1.67	-	+	
93	PI 34:1	M+H	837.5452	837.5488	4.30	+	-	
94	SM 42:1	M+Na	837.6785	837.6820	4.18	+	+	2.63
95	PC 38:1	M+Na	838.6315	838.6296	2.27	+	+	0.96
96	SM 44:1	M+H	843.7341	843.7313	3.32	+	-	
97	PI 35:4	M+H	845.5150	845.5175	2.96	+	-	
98	TG 50:2	M+Na	853.7249	853.7256	0.82	+	-	
99	PI 36:4	M+H	859.5307	859.5331	2.79	+	-	
100	PI 36:2	M+H	863.5612	863.5644	3.71	+	-	
101	PC 42:5	M+H	864.6508	864.6477	3.59	-	+	
102	PI 36:1	M+H	865.5765	865.5801	4.16	+	-	1.78
103	SM 44:1	M+Na	865.7164	865.7133	3.58	+	-	
104	TG 52:4	M+Na	877.7240	877.7256	1.82	+	-	
105	TG 54:6	M+H	879.7450	879.7436	1.59	+	-	

106	TG 52:2	M+Na	881.7559	881.7569	1.13	+	-
107	TG 52:1	M+Na	883.7733	883.7725	0.91	+	-
108	PI 38:4	M+H	887.5609	887.5644	3.94	+	-
109	PI 38:3	M+H	889.5789	889.5801	1.35	+	-
110	PI 38:2	M+H	891.5932	891.5957	2.80	+	-
111	PI 38:1	M+H	893.6088	893.6114	2.91	+	-
112	PI 40:4	M+H	915.5941	915.5957	1.75	+	-
113	PI 40:3	M+H	917.6088	917.6114	2.83	+	-
114	PI 40:2	M+H	919.6243	919.6270	2.94	+	-
115	PI 40:1	M+H	921.6393	921.6427	3.69	+	-
116	PI 42:5	M+H	941.6110	941.6114	4.25	+	-
117	PI 42:3	M+H	945.6417	945.6427	1.06	+	-
118	PI 42:2	M+H	947.6556	947.6583	2.85	+	-
119	PI 42:1	M+H	949.6702	949.6740	4.00	+	-

151

Table S7 Information of endogenous metabolites and lipids in negative ionization mode detected by MALDI and MALDI-2 MSI. Symbols, “+” and “-”, meant the meaningful and unmeaningful spatial distributions of endogenous molecules in breast CCS, respectively. Fold changes were the average intensities of metabolites in M2 divided by those in M1. Five biological replicates were contained in each group.

No	Name	Ion adduct	Theoretical *m/z*	Experimental *m/z*	|Δ ppm|	M2	M1	Fold change (M2/M1)
1	Inositol cyclic phosphate	M－H	241.0108	241.0119	4.46	+	+	1.05
2	Vaccenic acid	M－H	281.2479	281.2486	2.54	+	+	0.65
3	Stearic acid	M－H	283.2632	283.2642	3.67	+	+	0.40
4	Eicosatrienoic acid	M－H	305.2489	305.2486	0.94	+	+	0.43
5	Eicosadienoic acid	M－H	307.2640	307.2642	0.78	+	+	0.47
6	Eicosenoic acid	M－H	309.2786	309.2799	4.18	+	+	0.55
7	Adenosine monophosphate	M－H	346.0566	346.0558	2.30	+	+	3.90
8	ADP	M－H	426.0224	426.0221	0.60	+	+	0.29
9	LPA 18:1	M－H	435.2506	435.2517	2.53	+	+	0.70
10	Adenosine triphosphate	M－H	505.9881	505.9885	0.75	-	+	
11	LPI 18:0	M－H	599.3174	599.3202	4.67	+	+	0.32
12	ADP-ribose 1',2'-cyclic phosphate	M－H	620.0197	620.0202	0.79	-	+	
13	PA 32:2	M－H	643.4319	643.4344	3.89	+	+	0.60
14	DG 40:10	M－H	659.4665	659.4681	2.43	+	+	2.25

15	PA 34:3	M—H	669.4531	669.4501	4.48	+	+	0.65
16	PA 34:2	M—H	671.4645	671.4657	1.79	+	+	0.89
17	PA 34:1	M—H	673.4786	673.4814	4.16	+	+	1.04
18	PA 36:3	M—H	697.4781	697.4814	4.73	+	+	0.87
19	PA 36:1	M—H	701.5107	701.5127	2.85	+	+	0.31
20	PA 38:3	M—H	725.5109	725.5127	2.48	+	+	0.89
21	PA 38:2	M—H	727.5269	727.5283	1.92	+	+	1.79
22	PA O-40:6	M—H	733.5209	733.5178	4.23	+	-	
23	PE 36:5	M—H	736.4903	736.4923	2.72	+	+	1.70
24	PE 36:4	M—H	738.5073	738.5079	0.81	+	+	3.02
25	PE 36:3	M—H	740.5268	740.5236	4.32	+	+	1.82
26	PE 36:2	M—H	742.5357	742.5392	4.71	+	+	3.09
27	PE 36:1	M—H	744.5527	744.5549	2.95	+	+	6.01
28	PG 34:2	M—H	745.5006	745.5025	2.54	-	+	
29	PG O-36:4	M—H	755.5236	755.5233	0.40	-	+	
30	PE 38:4	M—H	766.5355	766.5392	4.83	+	+	3.88
31	PE 38:2	M—H	770.5695	770.5705	1.30	+	+	2.91
32	PG 36:1	M—H	775.5469	775.5495	3.35	+	+	0.61
33	PI 30:0	M—H	781.4889	781.4873	2.05	-	+	
34	PA 44:7	M—H	801.5408	801.5440	3.00	-	+	
35	PI 32:1	M—H	807.5023	807.5029	0.74	-	+	
36	PI 32:0	M—H	809.5195	809.5186	1.11	-	+	
37	PG 40:7	M—H	819.5165	819.5182	2.07	-	+	
38	PI 34:2	M—H	833.5184	833.5186	0.24	-	+	

39	PI 34:1	M－H	835.5338	835.5342	0.48	+	+	0.15
40	PI 34:0	M－H	837.5487	837.5499	1.43	+	+	0.13
41	PI 36:4	M－H	857.5211	857.5186	2.92	+	+	0.12
42	PI 36:3	M－H	859.5343	859.5342	0.12	+	+	0.10
43	PI 36:2	M－H	861.5480	861.5499	2.21	+	+	0.09
44	PI 36:1	M－H	863.5664	863.5655	1.04	+	+	0.08
45	PI 38:5	M－H	883.5348	883.5342	0.68	+	+	0.09
46	PI 38:4	M－H	885.5472	885.5499	3.05	+	+	0.08
47	PI 38:3	M－H	887.5641	887.5655	1.58	+	+	0.20
48	PI 38:2	M－H	889.5797	889.5812	1.69	+	+	0.09
49	PI 38:1	M－H	891.5939	891.5968	3.25	-	+	
50	PI 40:5	M－H	911.5630	911.5655	2.74	+	+	0.08
51	PI 40:4	M－H	913.5797	913.5812	1.64	+	+	0.12

Table S8 Information of significantly changed lipids and metabolites identified by MALDI-MSI. Letters, "N" and "P", refer to the "negative mode" and "positive mode", respectively. Statistical comparison of nine sections from three cell spheroids in each group was performed by using the paired t test.

No	Name	Detection mode	Fold change (Exp/Con, mean ± SD)	*p* value
1	PC(28:0)	P	1.68 ± 0.78	0.041
2	PC(30:1)	P	1.64 ± 0.72	0.035
3	PC(30:0)	P	1.53 ± 0.43	0.020
4	PC(32:2)	P	1.87 ± 0.94	0.026
5	PC(34:1)	P	1.81 ± 0.23	<0.001
6	PC(32:1)	P	1.85 ± 0.52	0.001
7	PC(34:2)	P	2.03 ± 0.53	<0.001
8	PC(36:2)	P	2.16 ± 0.37	<0.001
9	PC(36:1)	P	2.05 ± 0.32	<0.001
10	PC(38:4)	P	1.92 ± 0.28	<0.001
11	PC(38:3)	P	1.79 ± 0.26	<0.001
12	PC(38:2)	P	1.77 ± 0.26	<0.001
13	PC(42:5)	P	1.49 ± 0.23	<0.001
14	TG(48:1)	P	1.48 ± 0.39	0.007
15	TG(50:2)	P	1.64 ± 0.48	0.004
16	TG(52:4)	P	1.59 ± 0.49	0.007
17	TG(54:6)	P	1.69 ± 0.51	0.003

18	TG(52:2)	P	1.64 ± 0.48	0.003
19	TG(52:1)	P	1.56 ± 0.47	0.007
20	ATP	N	1.74 ± 0.63	0.030
21	ADP	N	1.79 ± 0.50	0.013
22	PE(16:0/20:4)	N	1.61 ± 0.27	0.0025
23	PE(18:1/18:2)	N	1.71 ± 0.31	0.0024
24	PE(18:0/18:2)	N	1.72 ± 0.34	0.0021
25	PE(18:0/18:1)	N	1.27 ± 0.13	0.0003
26	PE(18:0/20:4)	N	2.05 ± 0.43	0.0003
27	PI(16:0/16:1)	N	1.71 ± 0.51	0.0165
28	PI(18:1/18:2)	N	1.45 ±0.47	0.0458
29	PI(18:1/18:1)	N	1.86 ± 0.61	0.0070
30	PI(18:0/18:1)	N	2.02 ± 0.81	0.0104
31	PI(18:1/20:4)	N	1.84 ± 0.54	0.0035
32	PI(18:0/20:3)	N	2.46 ± 1.04	0.0023
33	PI(18:0/20:4)	N	2.19 ± 0.89	0.0035
